# Fermented sugarcane juice-derived probiotic *Levilactobacillus brevis* RAMULAB54 enhances lipid metabolism and glucose homeostasis through PPAR-γ activation

**DOI:** 10.3389/fmicb.2024.1502751

**Published:** 2025-01-29

**Authors:** V. B. Chandana Kumari, Ramith Ramu, Sujay S. Huligere, Shashank M. Patil, Shivasharanappa Nayakvadi, Sharath Bijoor, Uma Venkateswaran Manjappara, Mohammad Z. Ahmed, Ling Shing Wong

**Affiliations:** ^1^Department of Biotechnology and Bioinformatics, JSS Academy of Higher Education and Research, Mysore, Karnataka, India; ^2^ICAR-National Institute of Veterinary Epidemiology and Disease Informatics (NIVEDI), Bengaluru, Karnataka, India; ^3^Department of Plant Cell Biotechnology, CSIR-Central Food Technological Research Institute (CFTRI), Mysore, India; ^4^Department of Biochemistry, CSIR-Central Food Technological Research Institute (CFTRI), Mysore, India; ^5^Department of Pharmacognosy, College of Pharmacy, King Saud University, Riyadh, Saudi Arabia; ^6^Faculty of Health and Life Sciences, INTI International University, Nilai, Malaysia

**Keywords:** gut microbiota, PPAR-γ modulation, obesity, diabetes, hyperlipidemia, hyperglycemia

## Abstract

The gut microbiota plays a significant role in metabolic disorders such as diabetes and obesity, with the peroxisome proliferator-activated receptor gamma (PPAR-γ) being a key regulator in adipogenesis and glucose metabolism. This study is a novel approach that explores the antihyperglycemic and antihyperlipidemic effects of *Levilactobacillus brevis* RAMULAB54 (LB13243), isolated from fermented sugarcane juice. *LB13243* was cultured for SEM imaging, and its supernatant (LBR54) was analyzed. Organic acid interactions with PPAR-γ were evaluated via molecular docking, while cytotoxicity and adipocyte differentiation in 3T3-L1 cells were tested using MTT assays, Oil Red O staining, triglyceride quantification, and qRT-PCR. *In vivo*, male Wistar rats in hyperlipidemic and streptozotocin-induced hyperglycemic models were treated with *LB13243* for 4 weeks, followed by analysis of food intake, body weight, serum glucose, lipids, and histopathology. *LB13243* inhibited carbohydrate-hydrolyzing enzymes and showed an organic acid profile. *In silico*, hydroxycitric acid had similar binding to PPARγ as rosiglitazone (binding energy:−8.4 kcal/mol vs.−8.3 kcal/mol), with greater stability (RMSD: 1.2 Å vs. 1.7 Å). Pharmacokinetics indicated moderate GI absorption (20%) and low toxicity for hydroxycitric acid. LBR54 did not affect 3T3-L1 cell viability but reduced lipid accumulation by 13% and triglycerides by ≤ 44 mg/dL. qRT-PCR revealed upregulation of PPAR-γ and C/EBP-α, and downregulation of FAS, suggesting modulation of adipogenesis. *In vivo*, LB13243 reduced food intake, weight gain, and normalized organ weights in hyperlipidemic rats, while improving glucose levels and lipid profiles in hyperglycemic models. Histopathology showed improved tissue structure, indicating LB13243's potential to reduce hyperglycemia and hyperlipidemia by modulating lipid metabolism and inflammation. LB13243's modulation of PPAR-γ suggests it as a promising natural option for managing diabetes and hyperlipidemia. This study also highlights LB13243's distinctive capability to modulate PPAR-γ through its organic acids, particularly hydroxycitric acid, providing novel insights into its therapeutic potential.

## 1 Introduction

Obesity is a complex health problem that affects millions globally and is defined by an excessive accumulation of body fat. It is commonly measured using body mass index (BMI), with a BMI of 30 or higher classified as obesity (Ortuño Sahagún et al., 2012). Over the past few decades, the prevalence of obesity has risen significantly, creating a major public health challenge worldwide (Wondmkun, 2020). This increase is driven by a combination of factors such as—poor dietary habits, sedentary lifestyles, and easy access to calorie-dense, processed foods (Ismawanti et al., 2019; Kumari et al., 2024a). Obesity is associated with several serious health risks, including type 2 diabetes (T2D), cardiovascular disease, and dyslipidemia (Al-Goblan et al., 2014).

The treatment of obesity-related diabetes has focused on pharmaceutical approaches, with PPAR-γ agonists playing a crucial role. These drugs, including rosiglitazone and pioglitazone, enhance insulin sensitivity and regulate glucose and lipid metabolism (Ilavenil et al., 2015). PPAR-γ is essential for adipocyte differentiation, lipid storage, and the regulation of genes involved in fat metabolism. However, the use of these drugs is often limited due to side effects such as weight gain and increased cardiovascular risk (Muhlhausler et al., 2009; Huligere et al., 2022).

Therefore, there is a growing demand for safer and more effective treatments to address both prediabetes and obesity.

In recent years, the role of gut microbiota in obesity and metabolic diseases has gained attention, with probiotics emerging as potential therapeutic agents. Probiotics, particularly strains of *Lactobacillus*, have been shown to modulate metabolism, influence insulin sensitivity, and reduce inflammation (Sreepathi et al., 2022). Research suggests that probiotics may also interact with key metabolic pathways, including those regulated by PPAR-γ, offering a novel mechanism for managing obesity-related conditions (Iatcu et al., 2022). For instance, *Lactobacillus plantarum* has demonstrated the ability to enhance adipocyte differentiation and improve glucose uptake, suggesting its utility in managing T2D (Ilavenil et al., 2015).

In addition to probiotics, certain natural compounds, such as those derived from sugarcane, show promise in managing metabolic diseases. Recent studies have identified bioactive peptides and polyphenols in sugarcane that inhibit key enzymes involved in carbohydrate metabolism, such as α-glucosidase and α-amylase (Abduldileep et al., 2020; Martiz et al., 2023; Kumari et al., 2024b). These compounds may help regulate blood glucose levels and improve insulin sensitivity, offering an alternative to traditional pharmaceutical approaches (Ji et al., 2019). The study also identifies “schaftoside,” a flavonoid compound found in sugarcane leaves, as a potent inhibitor of Dipeptidyl peptidase-IV (DPP-IV), α-glucosidase, and α-amylase, showing significant inhibitory effects against these enzymes (Kan et al., 2023). Previous studies prove the presence of probiotics in sugarcane processing streams (Nel et al., 2020), sugarcane bagasse (Pattnaik et al., 2021), and fermented sugarcane juice (Kumari et al., 2023). Lactic acid bacteria (LAB), in particular *Lactobacillus* spp., can inhibit the activity of the enzymes α-glucosidase and α-amylase. *Lactobacillus* spp. can slow carbohydrate digestion during intake and absorption by blocking these enzymes. Reductions in blood sugar levels (hypoglycemia), better glucose management, increased pancreatic function, decreased insulin resistance, and a reduction in related oxidative damage are only a few of the positive benefits of this delay in carbohydrate metabolism (Kumari et al., 2022; Huligere et al., 2024). LAB can produce various organic acids such as lactic acid, acetic acid, propionic acid, butyric acid, hydroxycitric acid, malic acid, succinic acid, and citric acid, which play key roles in metabolism and health benefits (Kumari et al., 2023). Certain natural compounds, such as hydroxycitric acid, have shown potential in managing metabolic diseases. hydroxycitric acid, a natural organic acid derived from fermented foods and plants, has been widely studied for its lipid-lowering and glucose-regulating effects. By interacting with nuclear receptors like PPAR-γ, hydroxycitric acid influences adipogenesis, lipid storage, and inflammatory pathways, offering therapeutic potential for managing metabolic disorders (Arefhosseini et al., 2022; Verrelli et al., 2022).

Given the potential of probiotics and natural compounds in managing metabolic disorders, this study focuses on the strain *Levilactobacillus brevis* 13243 (*LB13243*). Preliminary research suggests that *LB13243* exhibits inhibitory effects on enzymes like α-glucosidase and α-amylase, which are involved in glucose metabolism (Kumari et al., 2023). Additionally, this strain may influence PPAR-γ signaling, offering a targeted approach for addressing hyperlipidemia and hyperglycemia in animal models. This study aims to investigate the *in silico, in vitro*, and *in vivo* effects of *LB13243* on adipocyte function and metabolic regulation. By exploring the potential of *LB13243* to modulate PPAR-γ activity, the research seeks to develop novel therapeutic strategies for obesity-related metabolic diseases.

## 2 Methodology

### 2.1 Preparation of *Levilactobacillus brevis* RAMULAB54 and SEM imaging

In our earlier research, we identified *Levilactobacillus brevis* RAMULAB54, a strain that exhibited promising antidiabetic properties and was isolated from fermented sugarcane juice. For the current study, we prepared the cell-free supernatant of *Levilactobacillus brevis* RAMULAB54, referred to as LBR54, using the method described by Kumari et al. (2023). The organic acids determined from the LCMS data of our previous study were reused to conduct *in silico* analysis in this research. This choice was made because the LCMS results were comprehensive and aligned with the objectives of this study (Kumari et al., 2023). However, to maintain the relevance and viability of the strains for further investigations, fresh cultures were prepared from frozen stocks at each stage of the study. This ensured the integrity of the experimental conditions and an accurate assessment of the strain's properties.

The preparation of the strain was carried out according to the protocol by Kang et al., which involved specific steps to optimize cell growth and ensure consistency for downstream applications. Briefly, the strain was cultured in MRS broth at 37°C, harvested during the log phase, and prepared for further analysis, including cell-free supernatant collection. Additionally, scanning electron microscopy (SEM) was performed using an EVO LS 15 (Carl Zeiss, Germany) at the Institute of Excellence, University of Mysore. This step provided critical insights into the surface morphology of the strain. The SEM analysis confirmed the rod-shaped structure characteristic of the *Levilactobacillus* genus, offering further evidence of its identity and potential viability for therapeutic use (Huligere et al., 2022).

### 2.2 *In silico* studies

To understand the binding interactions and stability of the organic acids inside the active site of the PPARγ protein, molecular docking, and molecular dynamics simulations were performed according to the previous studies by authors (Patil et al., 2021a, 2022b). For the molecular docking, the 3D structures of the organic acids were retrieved from the PubChem database and were 3D-modeled using ACD ChemSketch (Patil et al., 2022a). Similarly, the PPARγ protein molecule was retrieved from RCSB PDB (PDB ID: 3DZY) and was prepared by removing the heteroatoms using AutoDock Tools 1.5.6. (Martiz et al., 2022c). The protein and ligand molecules were converted to PDBQT format with Kollman and Gasteiger charges and AutoDock 4 atom type. The protein was kept rigid and was docked with the ligands using AutoDock Vina 1.2. (Martiz et al., 2022b,a). The results were analyzed by extracting binding snapshots using Biovia Discovery Studios Client 2021 (Patil et al., 2021b). The best protein-ligand complex and protein-rosiglitazone complexes were submitted to molecular dynamics simulation using GROMACS 2018.1 (Sajal et al., 2022). The complexes were added with CHARMM36 forcefield and ligand topology was obtained with SwissParam. The complexes were placed in a box with a 10 Å diameter with 0.15 M NaCl salt concentration and TIP3 water model. The NPT and NVT calculations were completed and the systems were equilibrated. Further, the simulation was performed for 100 ns. The trajectories obtained at the end of the simulation were plotted and analyzed using XMGRACE 5.1 (Maradesha et al., 2022b,a, 2023). The SMILES formats of the compound's rosiglitazone and hydroxycitric acid were taken and used to analyze the pharmacokinetics properties using SWISS ADME, with the criteria set according to the previous study of the authors (Simha et al., 2023).

### 2.3 3T3-L1 cell lines assay

#### 2.3.1 MTT cell viability assay

Cell viability MTT assay was conducted to assess **LBR54′**s cytotoxicity in 3T3-L1 preadipocytes., following the method described by Kim and Kong (2010). In brief, 3T3-L1 preadipocyte Cells were seeded at 10^5^ cells/mL in a 96-well plate, incubated for 24 h, and treated with LBR54 (10–100 μg/mL) for another 24 h. Rosiglitazone (RSG) served as a positive control, tested at 20–100 μg/mL (55.97–279.87 μM). The range of RSG concentrations was selected based on its reported effects on adipocyte differentiation and potential cytotoxicity at higher doses. Untreated cells served as controls. After treatment, MTT solution (5 mg/mL) was added and incubated for 4 h. Formazan crystals were dissolved in DMSO, and absorbance was measured at 540 nm using a Multiskan FC microplate reader (Thermo Scientific™, India). IC50 values were calculated using AAT Bioquest's online tool and validated through triplicate experiments (AAT Bioquest, Inc., 2024; Manimohan et al., 2024).

#### 2.3.2 Adipocyte differentiation

3T3-L1 cells (Passage No. 9) were sourced from the National Center for Cell Science, Pune, India. They were seeded at 5 × 10^5^ Cells/well) in 6-well culture plates with DMEM (Dulbecco's Modified Eagle Medium) containing 10% FBS (Fetal Bovine Serum) and incubated for 48 h until they reached approximately 70% confluency. The medium was then replaced with fresh DMEM containing 0.35 mM 3-isobutyl-1-methylxanthine, 25 μM dexamethasone, and 10 μg/mL insulin for the next 48 h. Following this, the medium was replaced every 48 h with DMEM containing 10 μg/mL insulin up to day 14 (Kim and Kong, 2010). For the first 3 days of differentiation, cells were treated with vehicle control [Phosphate Buffered Saline (PBS)] or LBR54 at concentrations of 10, 25, and 50 μg/mL. The intracellular lipid content was extracted and quantified at the end of the 14-day differentiation period.

#### 2.3.3 Qualitative and quantitative analysis of adipocyte differentiation by ORO Staining and triglycerides analysis

After 14 days of incubation, the cells were subjected to Oil Red O (ORO) staining to assess lipid content. Initially, cells were washed twice with PBS and fixed with 10% paraformaldehyde. Following fixation, cells were washed with 60% isopropanol and stained with ORO at room temperature. Excess dye was removed by washing with PBS, followed by a rinse with triple-distilled water (Lee et al., 2015). To prevent dehydration, the cells were overlaid with PBS. Morphological features were visualized and documented using a microscope and ProgRes^®^ CapturePro 2.7 software.

For quantitative analysis, triglyceride (TG) content in differentiated 3T3-L1 cells was measured as follows: cells were scraped into Tris-HCl buffer with EDTA (0.416 mg/mL, pH 7.5), then subjected to sonication and centrifugation. Lipids were extracted using Folch et al. (1951)'s method. Triglyceride levels in the extracted lipids were determined using a triacylglycerol assay kit (Agape diagnostic kits, Kerala, India).

#### 2.3.4 RNA extraction

Total RNA was extracted from 3T3-L1 adipocytes using the TRIzol reagent, as previously described (Lee et al., 2015). RNA concentration and purity were assessed by measuring the optical density ratio (A260/A280) with a Nanodrop^®^ ND-1000 spectrophotometer (NanoDrop Technologies in Wilmington, DE). Only RNA samples with an A260/A280 ratio >1.8 were selected for subsequent cDNA synthesis. Extracted RNA was stored at −80°C for long-term preservation. cDNA synthesis was performed using a commercial kit from Thermo Fisher Scientific (Bengaluru, India), following the manufacturer's instructions.

#### 2.3.5 qRT-PCR analysis

The mRNA expression of transcription factors and adipogenesis-related genes in differentiated 3T3-L1 cells was analyzed using quantitative real-time polymerase chain reaction (qRT-PCR). Total RNA was extracted from the cells using a Trizol reagent (Thermo Fisher Scientific, Bengaluru, India). This RNA was then reverse-transcribed into complementary DNA (cDNA) with a reverse-transcription kit (Takara, Bengaluru, India). qRT-PCR was performed using the SYBR Green PCR master mix (Takara, Bengaluru, India). The primer sequences used for this analysis are listed in Supplementary Table 1.

The qRT-PCR conditions were set for 40 cycles: initial denaturation at 95°C for 30 seconds, followed by 40 cycles of denaturation at 95°C for 5 seconds, and annealing/extension at 60°C for 30 seconds. Gene expression levels were normalized to β-actin, a reference gene, to ensure accurate quantification. Relative gene expression was calculated using the ΔΔCt method. Data were analyzed with appropriate software to determine statistical significance and interpret the results.

### *2.4 In vivo* studies

#### 2.4.1 Animals

Male Wistar albino rats (150–180 g) were housed at the Animal Facility, JSS College of Pharmacy, JSS AHER, Mysuru - 570015 (CPCSEA Registration No: 155/PO/Re/S/1999/CPCSEA). They were kept in polypropylene cages with controlled temperature and humidity, on a 12-h light/dark cycle. Following a 1-week acclimation period, the rats were fed a standard diet with *ad libitum* access to food and water. All procedures adhered to the CPCSEA guidelines and received approval from the Institute Animal Ethical Committee (IAEC) at JSS College of Pharmacy, JSS AHER, Mysuru (Ethical Approval No: JSSAHER/CPT/IAEC/089/2021).

*Levilactobacillus brevis* RAMULAB54 was deposited with the Microbial Type Culture Collection and Gene Bank (MTCC) at the CSIR-Institute of Microbial Technology, Chandigarh, India, under catalog number *Levilactobacillus brevis* MTCC 13243 (*LB13243*). The morphological characteristics of *LB13243* were examined using a scanning electron microscope (SEM; Hitachi S-4200, Tokyo, Japan) as described by Kang et al. (2019). Cells were incubated at 37°C for 24 h, washed, suspended in 0.1 M PBS (pH 7.2), and fixed with 2.5% glutaraldehyde in PBS for 3 h. After a 12-h incubation in PBS, the cells were rinsed, dehydrated through a graded ethanol series (10%, 25%, 50%, 75%, 95%, and 100% v/v), and dried at room temperature.

#### 2.4.2 Anti-hyperlipidemic activity and anti-hyperglycemic activity

For the anti-hyperlipidemic study, Wistar male rats acclimated for 1 week were fed a high-fat diet for 8 weeks to induce hyperlipidemia, which was confirmed by elevated total cholesterol levels. The rats were divided into four groups (Table 1): (1) Normal control, (2) High-fat diet control, (3) *LB13243* treatment (10^8^ CFU/mL), and (4) Rosiglitazone treatment (0.7 mg/kg). *LB13243* and rosiglitazone were administered intragastrically daily for 4 weeks. After euthanasia, blood samples and tissues (liver, adipose tissue, pancreas, and intestine) were collected for analysis.

**Table 1:** Grouping of the Wistar albino rats for antihyperlipidemic and antihyperglycemic study.

**Study**	**Groups**	**Treatment**	**No. of animals**	**Duration of the experiment**
Antihyperlipidemic activity	1	Normal diet control	10	12 weeks
	2	High fat-fed control	10	
	3	High-fat-fed rats treated with *LB13243* (10^8^ CFU/mL)	10	
	4	High fat-fed rats treated with Positive control rosiglitazone (0.7 mg/Kg of body weight)	10	
Antihyperglycemic activity	5	Normal glycaemic control	15	12 weeks
	6	Hyperglycaemic control	15	
	7	Hyperglycaemic control treated with *LB13243* (10^8^ CFU/mL)	15	
	8	Hyperglycaemic control treated with Positive control metformin (0.1 mg/Kg of body weight)	15	

For the anti-hyperglycemic study, Wistar male rats acclimated for 1 week were given a high-fat diet for 8 weeks. Diabetes was induced by intraperitoneal injection of streptozotocin (35 mg/kg body weight) dissolved in 0.1 M citrate buffer (pH 4.5). Rats with fasting blood glucose (FBG) levels of 250 mg/dL or higher 72 h later were considered diabetic and included in the study. Rats with FBG levels ≥400 mg/dL were excluded. Diabetic rats were divided into four groups (Table 1): (1) Normal glycemic control, (2) Hyperglycemic control, (3) *LB13243* treatment (10^8^ CFU/mL), and (4) Metformin treatment. *LB13243* and rosiglitazone were administered intragastrically for 4 weeks. Metformin (positive control) was administered at 0.1 mg/kg. After euthanasia, blood samples and tissues (liver, adipose tissue, pancreas, and intestine) were collected and stored at −80°C for subsequent analysis.

#### 2.4.3 Food intake, weight gain, and organ weight and oral glucose tolerance test

Food intake and weight gain were monitored weekly throughout the experimental periods- 4 weeks for the Antihyperglycemic activity study and 8 weeks for the Antihyperlipidemic activity study. After completing these periods, necropsy was performed to measure the weights of various organs. Oral glucose tolerance tests (OGTT) were conducted at the end of the 8-week HFD study and the 4-week antihyperlipidemic study. Before administering glucose (200 g/L solution; 2 g/kg body weight), rats were fasted for 12–14 h. Blood samples were collected from the tail vein at 0, 30, 60, and 120 min post-glucose administration and analyzed using a glucometer (Glucometer Digital, Janaushadhi Kendra, Karnataka, India).

#### 2.4.4 Serum biochemical analysis

Serum samples were analyzed for triglycerides, total cholesterol, HDL, LDL, VLDL, total protein, uric acid, urea, creatinine, glucose, albumin, and SGPT levels. The analyses were performed using kits from Takara (Bengaluru, India), and the resulting data were subjected to statistical analysis.

#### 2.4.5 RNA extraction and quantitative real-time polymerase chain reaction analysis for evaluation of marker genes

Tissues from the liver, pancreas, intestine, kidney, and visceral adipose tissue (1 mg each) were homogenized using a tissue homogenizer. RNA was extracted from the homogenate with 1 mL of Trizol reagent (Thermo Fischer Scientific, Bengaluru, India), following the manufacturer's protocol. The extracted RNA was then reverse-transcribed into cDNA using a cDNA Synthesis Kit.

For qPCR, the diluted cDNA (1:25) was used as a template, along with SYBR Green Jumpstart Taq ReadyMix (Sigma-Aldrich, Bengaluru, India) and gene-specific primers. The qPCR was conducted using a thermal cycler (Takara, Bengaluru, India) with the following parameters: initial denaturation at 94°C for 3 min, followed by 40 cycles of denaturation at 94°C for 30 seconds, annealing at 60°C for 30 seconds, and elongation at 72°C for 30 seconds. Melt curve analysis was performed to verify primer efficiency and specificity.

Oligonucleotide primers were designed using consensus sequences from rat genes obtained from the National Center for Biotechnology Information (NCBI) and were generated using the Primer3 software (http://primer3.ut.ee/). These primers were synthesized by Juniper Life Sciences Pvt. Ltd., Bengaluru, India, and are detailed in Supplementary Table 2. Gene expression was normalized using β-actin as an endogenous control. Relative gene expression levels were calculated using the 2–ΔΔCt method, which represents the fold change in gene expression.

#### 2.4.6 Tissue processing and histopathological studies

At the end of the experiment, animals were euthanized using cervical dislocation. Blood was collected via cardiovascular puncture, then centrifuged at 1,000 rpm for 20 min at 4°C to separate the serum, which was stored at −20°C until further analysis. Tissues from the liver, pancreas, kidney, intestine, and adipose tissue were collected and immediately preserved in RNA Later (Sigma-Aldrich Pvt. Ltd., Bengaluru, India) to stabilize the RNA and prevent degradation. The samples were stored at 4°C until further processing for RNA extraction and analysis.

For histopathological analysis, liver, kidney, muscle, pancreas, and intestinal tissues were sectioned and stained with hematoxylin and eosin (H&E). The stained sections were examined using a bright-field microscope (Labomed, Burlington, NC, USA) at, ×20 & ×40.

### 2.5 Statistical analysis

All tests were performed in triplicate, and results are presented as mean ± standard error. One-way analysis of variance (ANOVA) was conducted to compare the groups, followed by Duncan's Multiple Range Test to determine specific differences between groups. Statistical analyses were performed using SPSS Software (Version 21.0, Chicago, USA). A *p*-value of ≤ 0.05 was considered statistically significant. Graphs were created using GraphPad Prism version 8.0 (GraphPad Software Inc.).

## 3 Results

### 3.1 *Levilactobacillus brevis* RAMULAB54 and SEM imaging

Our previous research identified *Levilactobacillus brevis* RAMULAB54 as a notable isolate from nine strains derived from fermented sugarcane juice, demonstrating substantial inhibition of carbohydrate-hydrolyzing enzymes (Kumari et al., 2023). Molecular analysis via LC-MS revealed a distinct organic acid profile, with hydroxycitric acid emerging as a key compound with potential therapeutic benefits. *In silico* studies indicated that hydroxycitric acid has a binding affinity for the enzyme inhibition site involved in carbohydrate hydrolysis, suggesting a possible mechanism of action.

Supplementary Figure 1 illustrates a structural examination of *Levilactobacillus brevis* RAMULAB54 using SEM, highlighting its morphological characteristics. The combination of enzyme inhibition assays, LC-MS profiling, and SEM imaging underscores the significant potential of *Levilactobacillus brevis* RAMULAB54. Despite these promising findings, further research is necessary to explore its therapeutic and biotechnological applications thoroughly. Continued investigation will enhance our understanding of microbial biochemistry and support the development of novel enzymatic inhibitors with applications in pharmaceuticals and food technology.

### 3.2 Molecular docking

During the molecular docking simulation, all the organic acids obtained from the LC-MS analysis of the LBR54 were found to get bound inside the binding pocket of the PPARγ protein, including hydroxycitric acid (Supplementary Figure 2). The binding affinity of the molecules ranged between −8.0 to −11.0 kcal/mol, with hydroxycitric acid being the lead molecule with the highest/most negative binding affinity (−11.0 kcal/mol). On the other hand, the control drug used rosiglitazone had a binding affinity of −12.0 kcal/mol. The results of the virtual screening of organic acids against PPARγ protein based on binding affinity, total number of interactions, and total number of hydrogen bonds are given in Supplementary Table 3.

Both the experimental compounds hydroxycitric acid and rosiglitazone were bound in the exact position of the co-crystallized ligand rosiglitazone, occupying the ligand-binding site (LBD) of the PPARγ protein. The core of the LBD lies between the H3, H5, and H7 domains of the Y-shaped receptor molecule (Chandra et al., 2008). While interacting with the residues of the PPARγ protein, hydroxycitric acid was bound with Ser289 (2.77 Å, 2.63 Å, and 2.70 Å), Tyr473 (1.97 Å), His323 (2.59 Å), His449 (3.74 Å), and Tyr327 (3.43 Å) hydrogen bonds. The compound was found to bind with the residues only with hydrogen bonds (Supplementary Figure 3). There were no other types of intermolecular bonds involved. This could be the reason behind its highest binding affinity toward the protein. However, it is believed that binding affinities increase by about one order of magnitude per hydrogen bond (Kawasaki et al., 2010). Complexes with more strong hydrogen bonds have a stronger effect on the formation of the complex and the docking result would be more accurate (Schaeffer, 2008).

The compound is bound in the exact position of the co-crystallized ligand rosiglitazone, occupying the ligand-binding site (LBD) of the PPARγ protein. The core of the LBD lies between the H3, H5, and H7 domains of the Y-shaped receptor molecule. Hydroxycitric acid binds to His323, one of the key catalytic residues of the LBD. The rosiglitazone bound with LBD through 12 intermolecular interactions with 8 of them being hydrogen bonds. The hydrogen bonds include Arg288 (3.33 Å), Tyr473 (2.49 Å), His323 (3.69 Å and 3.04 Å), His449 (3.09 Å), Gly284 (3.37 Å), and Cys285 (3.49 Å and 4.16 Å). Rosiglitazone had also bound with Met348 (5.33 Å) and Met364 (5.23 Å) through hydrophobic pi-sulfur bonds, and Leu330 (4.77 Å) and Ile341 (4.68 Å) through hydrophobic pi-alkyl bonds (Supplementary Figure 3). The binding mode of the experimental compound from our study rosiglitazone was similar to the co-crystallized agonist rosiglitazone (PDB ID: 3DZY) reported (Chandra et al., 2008).

According to Chandra et al. (2008), the co-crystallized agonist rosiglitazone binds with the residues Ile341, Ser289, Tyr473, Cys285, His449, His323, Leu330, Met364, Tyr328, and His449 which majorly constitute the PPARγ LBD (PDB ID: 3DZY). While binding with the PPARg LBD, hydroxycitric acid had 3 common binding residues Ser289, His323, and His449, which are also bound by co-crystallized agonist rosiglitazone (Chandra et al., 2008). Therefore, binding to these residues or in the vicinity of these residues would activate the catalytic activity of the protein, with a similar mechanism to rosiglitazone.

Similar outcomes were found in a study where 1,3-Diphenyl-2-Propanone was reported as a potential PPARγ agonist (Arulkumar et al., 2021). The ligand reported in this study was bound to the residues, Cys276, Gln277, Ser280, Tyr314, Met355, Leu456, and Tyr464, which are the part of PPARγ LBD (PDB ID: 3DZY). The ligand binding occurred within a similar region as observed in our study and in the findings of Chandra et al. (2008). where co-crystallized rosiglitazone was also reported to occupy the same ligand-binding domain (LBD). The observed interactions also align with findings from a previous study by Ashraf et al. (2021), which identified abscisic acid interactions within the ligand-binding domain (LBD) of PPARγ. Key residues involved in the interaction included Tyr250, Lys194, and Ala197, situated within the same binding site region where hydroxycitric acid, as identified in our study, was bound to the PPARγ LBD (PDB ID: 3DZY). A comparative account of the binding mode of these studies with reported PPARγ agonists has been given in Table 2.

**Table 2:** Comparative account of the binding mode of the reported PPARg agonists.

**Sl. No**.	**Ligand**	**Binding site**	**PPARg binding residues**	**References**
1	1,3-Diphenyl-2-Propanone	PPARγ LBD	Cys276, Gln277, Ser280, Tyr314, Met355, Leu456, and Tyr464,	Arulkumar et al., 2021
2	Rosiglitazone	PPARγ LBD	Ile341, Ser289, Tyr473, Cys285, His323, Leu330, Met364, Tyr328, and His449	Chandra et al., 2008
3	Abscisic acid	PPARγ LBD	Tyr250, Lys194, and Ala197	Ashraf et al., 2021

The *in vitro* outcomes from these studies strongly suggest that the above-mentioned compounds have significant PPARγ agonist activity, thus acting as a beneficial effect toward diabetic conditions. The binding mode of hydroxycitric acid strongly resembled that of the rosiglitazone, the co-crystallized PPARγ agonist. Rosiglitazone blocks PPARγ phosphorylation, preventing the suppression of insulin-responsive genes. It also increases PPARγ expression in renal tubular epithelial cells, offering protection against hydrogen peroxide-induced damage (Wei et al., 2022).

This confirms the ability of hydroxycitric acid, through its hydrogen bonds, to affect the receptor's DNA-binding properties (Chandra et al., 2008; Arulkumar et al., 2021). Therefore, hydroxycitric acid could act as a potential PPARγ agonist, which can be used as an antidiabetic drug in near future.

#### 3.2.1 Molecular dynamics simulation

The molecular dynamics (MD) simulations were performed to analyze the dynamic stability of the ligand molecule inside the binding pocket of the protein molecule. These simulations run for 100 ns resulting in the generation of MD trajectories that are divided as protein-ligand complex's root mean square division (RMSD) with the actual position of ligand binding site in the protein, root mean square fluctuations (RMSF) of the protein-ligand complex inside the binding site, the radius of gyration (Rg) that depicts the compactness of the protein-ligand complex throughout the simulation, solvent accessible surface area (SASA) that defines the ability of the ligand molecule to occupy the surface area, and ligand hydrogen bonds (H-Bonds) that are continuously formed between the protein and ligand during the simulation.

The RMSD trajectories show that both the protein-hydroxy citric acid complex and protein-rosiglitazone complex attained stability over 30 ns, indicating the stability of both complexes inside the simulation box. The complexes equilibrated at 0.15 nm along with the PPARγ apo-protein (Supplementary Figure 4A). Since both the complexes follow the apo-protein in terms of stability and equilibration, they are believed to be stable throughout the simulation. The RMSF trajectories depict the fluctuation of both complexes along the apo-protein. The fluctuations of both the protein-hydroxy citric acid complex and protein-rosiglitazone complexes were found to be lower (ranging between 0.0 to 0.3 nm) than that of the apo-protein molecule (ranging between 0.0 to 0.35 nm), indicating the stability achieved upon the binding of the ligand (Supplementary Figure 4B). Similarly, the compactness of the ligand atoms involved in the simulation was found to be stable during the Rg analysis. Both the complexes with atoms were compactly bound (Supplementary Figure 4C). This compactness makes the surface area of the LBD decrease due to the binding of the ligand molecule. Therefore, the SASA of the protein-ligand complexes has decreased from 145 nm^2^ to 140 nm^2^ (Supplementary Figure 4D). The ligand hydrogen bond analysis shows that rosiglitazone formed a maximum of 5 hydrogen bonds with the protein LBD, whereas hydroxycitric acid made 4 hydrogen bonds (Supplementary Figure 4E). From the perspective of molecular docking, the complexes were stable and did not produce unstable fluctuations during the entire simulation period.

The MD trajectories obtained during the simulation followed the previous study where 1,3-Diphenyl-2-Propanone was reported as a potential PPARγ agonist (Arulkumar et al., 2021). However, in comparison with this study, the stability of hydroxycitric acid was found to be more, and the same was reflected in the MD trajectory plot analysis. Since rosiglitazone has also been reported with the same binding region, hydroxycitric acid could also be recognized as a potential PPARγ agonist (Chandra et al., 2008).

#### 3.2.2 Pharmacokinetics calculations

The pharmacokinetic analysis of rosiglitazone and hydroxycitric acid showed that both compounds could be consumed orally. However, rosiglitazone showed the possibility of drug-induced liver injury and AMES toxicity. This could be the result of continuous and prolonged usage of the drug. Recent reports also suggest that prolonged use causes abnormal deposits of fat. Also, the drug causes anemia, back pain, fatigue, headache, hypoglycemia, myalgia, and sinusitis (Bundhun et al., 2017). Though hydroxycitric acid has been predicted with moderate GI absorption, changing the drug concentration could help fix the right dose for oral administration. Therefore, *in vivo* studies and clinical trials need to be conducted. In addition, the drug showed CYP inhibition (Supplementary Table 4). The cytochrome P450 (CYP) enzyme family is responsible for the phase 1 metabolism of pharmaceuticals, herbal remedies, and toxic compounds in the environment. This enzyme system is highly important as it plays a crucial role in the breakdown of these substances. However, inhibition and induction of CYPs can lead to drug-drug interactions, affecting the efficacy and safety of pharmaceuticals (Hakkola et al., 2020). Therefore, the pharmacokinetics analysis indicates that hydroxycitric acid could be used as a potential PPARγ agonist, and could replace rosiglitazone and other TZDs in the near future as such.

### 3.3 3T3-L1 cell lines assay

#### 3.3.1 MTT cell viability assay

The results showed that LBR54 treatment did not significantly impact the viability of 3T3-L1 cells across the tested concentrations. The percent viability remained consistent, indicating that LBR54 did not exhibit noticeable cytotoxicity within the concentration range tested (Supplementary Figure 5A). This suggests that LBR54 is well-tolerated by 3T3-L1 cells, maintaining high cell survival rates.

In contrast, RSG treatment led to a concentration-dependent decrease in cell viability. At the lowest concentration of 20 μg/mL (55.97 μM), cells exhibited the highest viability of 61.91%. However, as RSG concentrations increased to 40 μg/mL (111.94 μM), 60 μg/mL (167.92 μM), 80 μg/mL (223.89 μM), and 100 μg/mL (279.87 μM), cell viability progressively decreased. This trend confirms the cytotoxic effects of RSG at higher concentrations, consistent with previous studies indicating RSG's potential cytotoxicity (Konno et al., 2020).

Previous studies evaluating *Lactobacillus* strains, such as *Lactobacillus plantarum* LMT1-48, *Lactobacillus sakei* KBL, and *Weissella koreensis* 521, found no cytotoxic effects on 3T3-L1 cells (Pi et al., 2014; KiBeom and GunSu, 2015; Choi et al., 2020). These findings suggest that probiotic extracts, including LBR54, are promising candidates for further investigation as non-toxic additives in medical and biotechnological applications.

#### 3.3.2 Adipocyte differentiation, qualitative and quantitative analysis of adipocyte differentiation by ORO staining and triglycerides analysis

Adipogenesis, the process where preadipocytes mature into adipocytes, is influenced by various growth factors, cytokines, and hormones (Kim and Kong, 2010). Studies by Jang et al. (2019) and Zhang et al. (2016) have shown that *Lactobacillus* spp. can inhibit adipocyte differentiation, highlighting its potential role in regulating adipose tissue metabolism. These findings contribute to our understanding of the complex interplay between gut microbiota, adipogenesis, and metabolic health.

**Figure 1:**
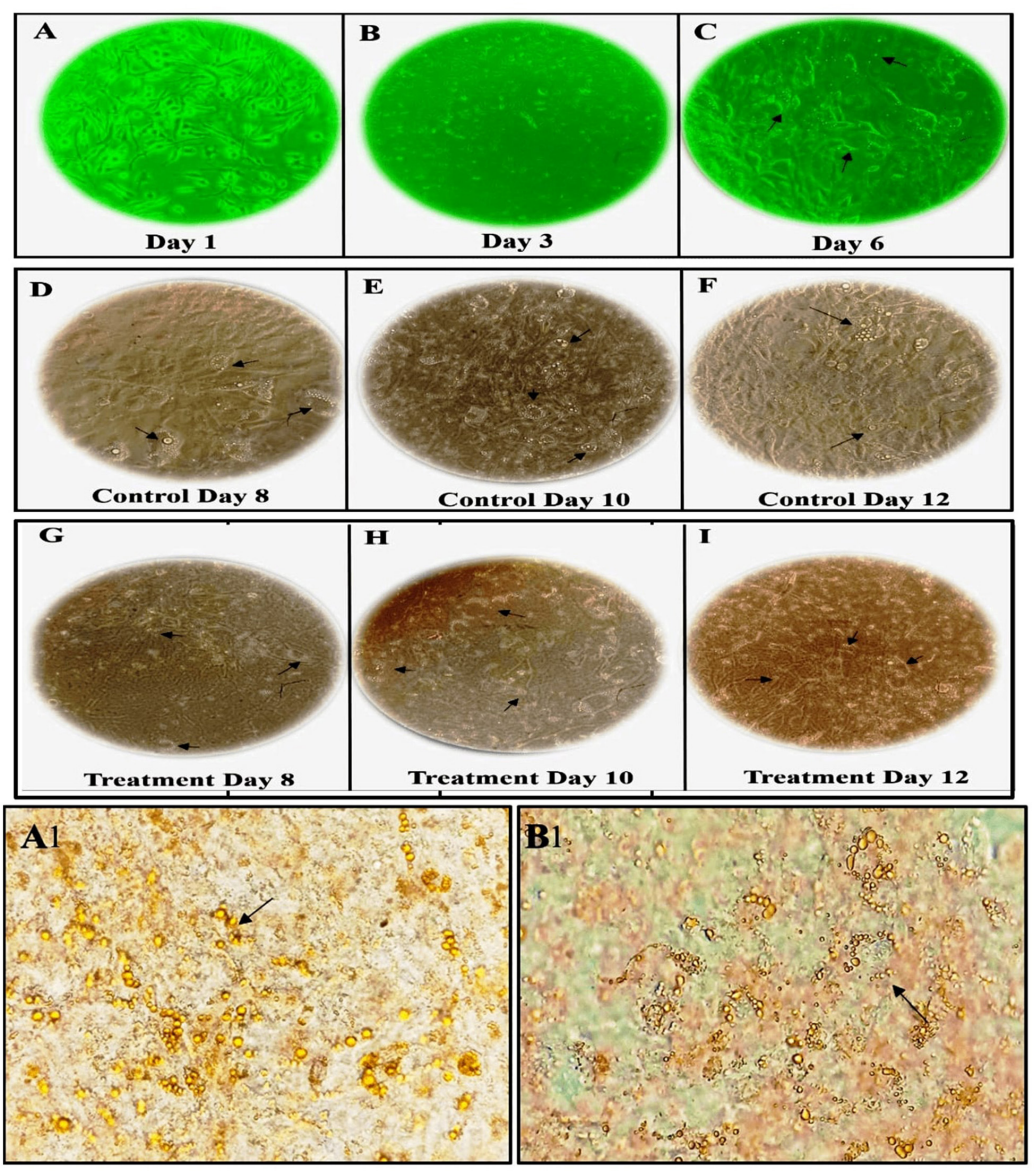
Morphological changes and Oil Red O staining of 3T3-L1 cells before and after adipogenic stimulation. **(A–C)** morphological features of 3T3-L1 cells from Days 1 to 6 (Pre-treatment), **(D–I)** morphological features of 3T3-L1 cells from Days 8 to 12 (Post-treatment), showing both control **(D–F)**, and LBR54-treated cells **(G–I)**. After adipogenic stimulation, lipid droplet accumulation occurred more rapidly in Control cells compared to treated 3T3-L1 cells (Black arrow). Oil Red O staining on Day 14 further illustrates the accumulation of lipid droplets (Black arrow) in **(A1)** control untreated cells compared to **(B1)** LBR54 treated 3T3-L1 cells.

In our study, we observed a dose-dependent reduction in lipid accumulation in 3T3-L1 cells treated with LBR54. Specifically, lipid accumulation decreased from 73.6% at 10 μg/mL to 60.5% at 75 μg/mL LBR54 (Supplementary Figure 5B). This suggests that LBR54 effectively inhibits lipid accumulation compared to the untreated control, indicating its potential in modulating adipogenesis. TG analysis further supported these findings, with LBR54 treatment resulting in a significant reduction in TG content. The TG levels decreased from 136.41 mg/dL in control cells to 92 mg/dL at the highest concentration of 75 μg/mL LBR54 (Supplementary Figure 5C). This reduction was consistent across concentrations, demonstrating the effective inhibition of TG accumulation by LBR54. The morphological features of the cells were documented from days 1 to 6 (Pre-treatment) (Figures 1A–C) and from days 8 to 12 (Post-treatment), with both control (Figures 1D–F) and LBR54 treatments (Figures 1G–I), using a microscope and ProgRes^®^ CapturePro 2.7 software (Figures 1A–I).

The ORO staining results on day 14 (Figures 1A1, B1) revealed that untreated control cells showed a significant increase in lipid droplets, indicating extensive lipid accumulation. In contrast, cells treated with LBR54 displayed fewer lipid droplets, with the reduction becoming more pronounced as the concentration of LBR54 increased. This dose-dependent decrease in lipid accumulation underscores LBR54′s potential to modulate adipogenesis by inhibiting lipid droplet formation. Specifically, the control group exhibited a substantial rise in lipid droplet formation (Figure 1A1), while LBR54-treated cells showed a marked decrease in lipid accumulation, with the degree of inhibition directly related to the concentration of LBR54 used (Figure 1B1). This highlights the effectiveness of LBR54 in regulating adipogenesis.

Our results align with previous studies, such as those by Lee et al., who reported decreased lipid accumulation in 3T3-L1 cells following treatment with fermented products of *L. mesenteroides* (Lee et al., 2014). In their research, Lee et al. *L. mesenteroides* and *L. plantarum* starters in the fermentation process of kimchi had an impact on the fatty acid and amino acid compositions. These changes in composition likely contributed to the observed reduction in lipid accumulation in 3T3-L1 adipocytes (Lee et al., 2015). In the study conducted by Oh et al., cell-free extracts derived from three newly identified strains of *L. plantarum* demonstrated cell non-toxicity at concentrations of 50 and 100 μg/mL (Oh et al., 2021).

Obesity's association with health and metabolic disorders is closely linked to the processes of adipocyte differentiation and TG accumulation in adipose tissue (Rizzatti et al., 2013). These results align with previous studies indicating the ability of certain treatments to inhibit adipogenesis and reduce lipid accumulation. For instance, Park et al. (2011) demonstrated that *L. plantarum* enhances glycerol release from 3T3-L1 adipocytes and reduces TG levels significantly. Similarly, Kim et al. (2018) reported that *L. plantarum* K10 inhibited adipocyte differentiation by 32% at 100 μg/mL.

#### 3.3.3 RNA Extraction and qRT-PCR analysis

**Figure 2:**
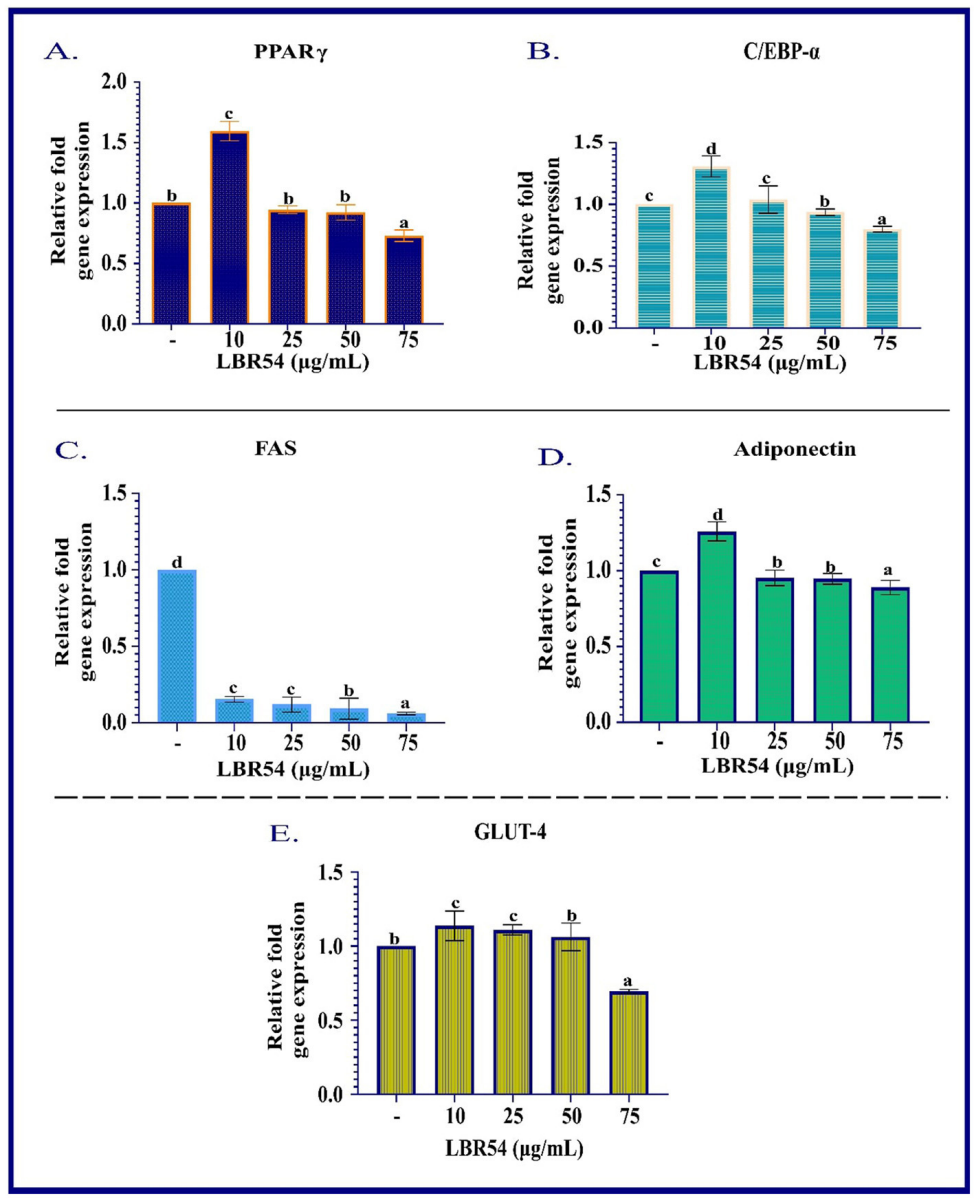
RNA Gene Expression Analysis in Differentiated 3T3-L1 Adipocytes. qRT-PCR was performed to assess the mRNA expression levels of **(A)** PPAR-γ, **(B)** C/EBP-α, **(C)** FAS, **(D)** adiponectin, and **(E)** GLUT-4 in differentiated 3T3-L1 cells. Gene expression was normalized to β-actin, and relative expression levels were calculated using the ΔΔCt method. Data are presented as mean ± SE from three independent biological replicates. Statistical significance was determined using one-way ANOVA followed by DMRT the superscripts (a–d) are significantly different (*p* ≤ 0.05) between groups.

The RNA extraction and qRT-PCR analysis of 3T3-L1 cells treated with LBR54 reveal that gene expression is influenced by the concentration of LBR54, highlighting complex regulatory effects. Figure 2 illustrates the concentration-dependent responses of several key genes involved in adipogenesis. At higher LBR54 concentrations (25 μL, 50 μL, and 75 μL), PPAR-γ expression was upregulated. This suggests that LBR54 may initially stimulate PPAR-γ expression, thereby promoting adipocyte differentiation. However, the elevated PPAR-γ levels at higher concentrations could indicate either a positive feedback mechanism or a differential regulatory effect of LBR54 (Figure 2A). Similar to PPAR-γ, C/EBP-α showed increased expression at higher concentrations of LBR54 (25 μL, 50 μL, and 75 μL). This suggests that LBR54 potentially promotes early adipocyte differentiation through upregulation of C/EBP-α (Figure 2B). Our findings highlight the concentration-dependent effects of LBR54 on gene expression in 3T3-L1 cells, underscoring the complexity of its impact on adipogenesis. The initial upregulation of PPAR-γ and C/EBP-α at higher concentrations suggests that LBR54 might stimulate adipocyte differentiation, with potential positive feedback or alternative regulatory mechanisms at play at higher doses (Kim et al., 2020; Sreepathi et al., 2023; Xu et al., 2024).

FAS, crucial for fatty acid synthesis, was consistently downregulated across all LBR54 concentrations. This indicates that LBR54 may inhibit fatty acid synthesis, which could affect lipid metabolism and adipogenesis. The consistent downregulation of FAS across all tested concentrations of LBR54 suggests an inhibition of fatty acid synthesis (Figure 2C). This could have implications for lipid metabolism and overall adipogenesis, aligning with previous studies that demonstrated the impact of various compounds on FAS expression and fatty acid synthesis (Kong et al., 2010; Ilavenil et al., 2015; Worthmann et al., 2024).

The expression of Adiponectin was upregulated by LBR54 treatment, suggesting a potential enhancement in glucose and lipid metabolism, given Adiponectin's role in improving insulin sensitivity and metabolic balance (Figure 2D). The upregulation of Adiponectin by LBR54 indicates a beneficial effect on metabolic processes, potentially enhancing insulin sensitivity and regulating glucose and lipid metabolism, as supported by prior research (Kong et al., 2010).

GLUT-4 exhibited upregulation at lower LBR54 concentrations (10 μL and 25 μL) and downregulation at higher concentrations (50 μL and 75 μL) (Figure 2E). This indicates that LBR54′s effect on GLUT-4 is concentration-dependent, influencing glucose uptake and possibly adipocyte function. The observed regulation of GLUT-4 expression by LBR54, with initial upregulation followed by downregulation at higher concentrations, suggests a nuanced effect on glucose uptake. This complex regulation may reflect alterations in glucose metabolism or adipocyte function, as seen in previous studies of glucose transporter regulation (Kord et al., 2020).

Comparatively, studies by Park et al. and Kim et al. on KY1032-CE and *Lactobacillus fermentum* cell-free extracts respectively, have demonstrated similar dose-dependent effects on lipid accumulation and adipogenesis, further validating the influence of treatment concentration on adipocyte function (Park et al., 2011; Kim et al., 2020). The complex regulation of GLUT-4 and the differential impact on adipogenesis observed in our study may provide insights into the broader implications of LBR54 treatment on metabolic processes. LBR54′s concentration-dependent effects on gene expression in 3T3-L1 cells emphasize its complex role in adipogenesis, affecting key transcription factors, lipid metabolism, and glucose uptake.

### *3.4 In vivo* studies

#### 3.4.1 Food intake and weight gain

Hyperlipidemia, defined by increased levels of cholesterol and triglycerides, represents a significant global health challenge linked to various cardiovascular conditions (Ghatani et al., 2022). Managing hyperlipidemia effectively often involves a multifaceted approach that includes lifestyle adjustments, dietary modifications, and, when necessary, medication. Key strategies include regular exercise, maintaining a healthy body weight, and adhering to a diet low in saturated and trans fats to manage lipid levels and mitigate associated health risks (Yan et al., 2019; Huligere et al., 2023).

In our study focusing on antihyperlipidemic food intake and weight gain study, we observed that groups on an HFD (Group 2) had notably higher food intake compared to the normal control group (Group 1). However, after 4 weeks of treatment with potential HFD-treated *LB13243* (Group 3), these groups exhibited a slight decrease in food consumption. Furthermore, while initial weight gain was observed in the HFD groups (Group 2), those treated with *LB13243* (Group 3) showed a gradual weight reduction, unlike the HFD treated with rosiglitazone control (Group 4), which continued to gain weight throughout the study.

**Figure 3:**
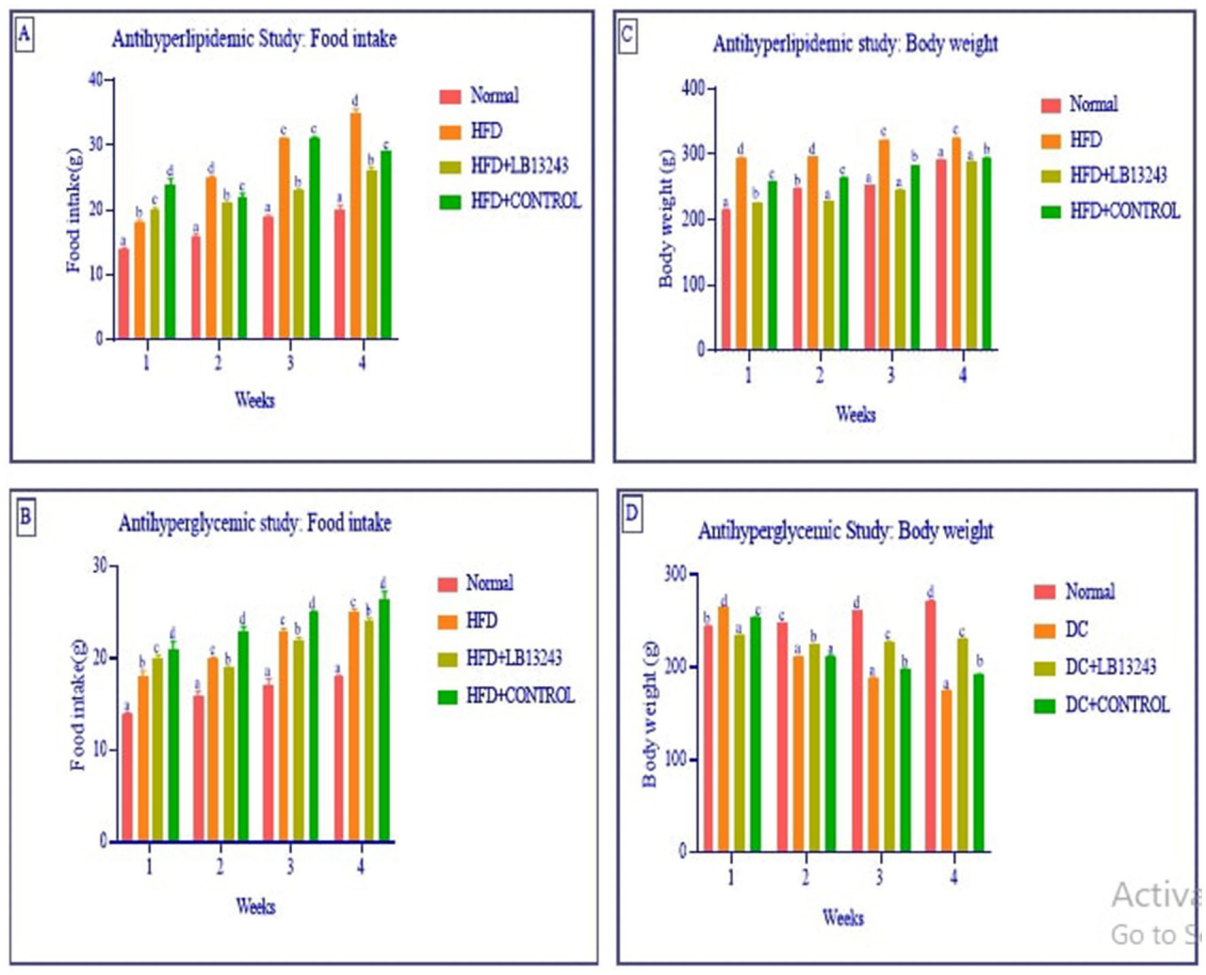
**(A, B)** Food intake and **(C, D)** body weight in the anti-hyperlipidaemic and anti-hyperglycaemic studies. In the anti-hyperlipidaemic study, each group consisted of *n* = 10 animals, while in the anti-hyperglycaemic study, each group consisted of *n* = 15 animals. Data are presented as mean ± SE. Statistical significance was determined using one-way ANOVA followed by DMRT. Comparisons were considered with the superscripts (a–d) significant for *p*-values ≤ 0.05.

Streptozotocin is a naturally derived compound with dual properties—it acts as a broad-spectrum antibiotic and also has cytotoxic effects. Researchers often use it to induce diabetes in rats because of its specific toxicity toward the insulin-producing β-cells of the pancreas (Srihari et al., 2013). In antihyperglycemic food intake and weight gain study, the induction of diabetes using Streptozotocin resulted in weight loss in the rats when compared to the normal glycaemic control (Group 5). The hyperglycaemic control rats (Group 6) showed characteristic symptoms of diabetes, including increased urination, heightened food intake, and elevated water consumption. These symptoms are commonly associated with uncontrolled diabetes in rodents. In contrast, the hyperglycaemic groups treated with *LB13243* (Group 7) did not exhibit significant differences in body weight when compared to the diabetic control rats treated with metformin. The changes in food intake and body weight are detailed in Figures 3A–D, respectively.

In prior studies, the group of rats fed an HFD experienced significant weight gain, higher blood glucose levels, and elevated cholesterol levels (Lian et al., 2007; Srihari et al., 2013; Zulkawi et al., 2018). The study conducted by Melhorn et al. indicates that the consumption of a high-fat diet can lead to significant and rapid changes in meal patterns. These altered eating habits, in turn, contribute to the development of adiposity, which refers to the accumulation of body fat and, potentially, obesity (Melhorn et al., 2010). *Lactobacillus plantarum* GKM3 supplementation can play a beneficial role in mitigating the harmful effects of a high-fat diet. By promoting more balanced metabolic processes and potentially modulating the body's response to dietary fats, this probiotic supplementation may aid in maintaining a healthier weight and reducing the risks associated with high-fat diets, such as obesity and related metabolic disorders (Hsu et al., 2019). *Lactobacillus gasseri* BNR17, a probiotic strain isolated from human breast milk, has demonstrated the ability to inhibit weight gain, highlighting its potential as an anti-obesity agent (Crovesy et al., 2017). *Lactobacillus plantarum* LB818 shows potential in preventing fat accumulation in adipose tissue and hepatic steatosis in obese mice. LB818 also can restore gut microbiota in diet-induced obese mice, indicating its potential as a therapeutic intervention for obesity and related metabolic disorders (Hussain et al., 2020).

#### 3.4.2 Organ weight and oral glucose tolerance

Excess adiposity is associated with elevated sympathetic activity in the kidneys and skeletal muscle. This increased sympathetic activity can lead to metabolic dysfunction, including insulin resistance and diabetes, as well as early organ dysfunction (Lin and Li, 2021). In the antihyperlipidemic study, there are significant variations in organ weights across the different treatment groups. The Normal diet control group (Group 1) exhibits the lowest values for the heart, liver, pancreas, and adrenal glands, and its intestine weight is also among the lowest. Conversely, the High fat-fed control group (Group 2) shows the highest organ weights, including the heart (1.53 ± 0.98 g), liver (11.41 ± 1.52 g), and kidneys (2.19 ± 1.23 g), reflecting a marked increase compared to other groups. The High-fat-fed rats treated with *LB13243* (Group 3) present intermediate values, demonstrating a beneficial effect as this treatment appears to moderate the organ weights compared to the high-fat-fed control group, suggesting its potential to counteract some of the adverse effects of the high-fat diet. Meanwhile, the High fat-fed rats treated with Positive control rosiglitazone (Group 4) have higher liver and adrenal gland weights compared to the Normal diet control but lower intestine weight.

In the antihyperglycemic study, significant differences in organ weights are observed among the groups. The hyperglycaemic control group (Group 6) exhibits the highest weights in the liver, pancreas, and intestine, reflecting a pronounced impact of hyperglycemia on these organs. Conversely, the Normal glycaemic control group (Group 5) shows relatively lower values. The hyperglycaemic control treated with *LB13243* (Group 7) demonstrates a balanced profile, with intermediate organ weights suggesting that *LB13243* effectively moderates the impacts of hyperglycemia on organ health, positioning it as a promising treatment option. Meanwhile, the Hyperglycaemic control treated with Positive control metformin (Group 8) has the lowest heart and kidney weights but relatively high liver and intestine weights. This indicates that metformin may have a distinct impact on heart and kidney health compared to other treatments. However, in the groups that received supplementation with *LB13243*, the organ weights appeared to be normalized, as indicated by the data presented in Supplementary Table 5.

In a study by Archer et al., it was found that *Lactobacillus fermentum* strains demonstrated the ability to reduce inflammation in several tissues, including the liver, muscle, and adipose tissues. This suggests that these particular strains of *Lactobacillus fermentum* may have anti-inflammatory properties, which could be beneficial in mitigating inflammation-related health issues associated with a high-fat diet (Archer et al., 2021). Additionally, the introduction of *Lactobacillus plantarum* 9-41-A led to significant reductions in weight gain, liver and fat pad mass, and adipocyte size. These findings indicate that *Lactobacillus plantarum* 9-41-A supplementation may have a potential role in limiting weight gain and fat accumulation, particularly in adipose tissues. This could be beneficial in preventing obesity-related complications that may arise from a high-fat diet (Xie et al., 2011). Both studies demonstrate the potential benefits of using specific probiotic strains, *Lactobacillus fermentum*, and *Lactobacillus plantarum* 9-41-A, as interventions to address the negative effects of a high-fat diet on organ weight, inflammation, and adipose tissue expansion. In a study by Srihari et al., fermented tea did not cause any changes in body mass, and it also reduced the glycemic index. This suggests that fermented tea may have potential health benefits, particularly in terms of blood sugar regulation (Srihari et al., 2013). Furthermore, in a study conducted by Lee et al., supplementation with *Lactobacillus brevis* MJM60390 was found to reverse kidney damage caused by hyperuricemia. Hyperuricemia is a condition characterized by high levels of uric acid in the blood, and it can contribute to kidney damage. The results imply that *Lactobacillus brevis* MJM60390 supplementation may have a protective effect on kidney health, potentially counteracting the detrimental effects of hyperuricemia (Lee et al., 2022).

The OGTT is a test used to evaluate how effectively an individual can regulate blood glucose levels after consuming a specific amount of glucose orally. In the antihyperglycemic study, the OGTT test involves monitoring blood glucose levels at various time intervals, typically at 0, 15, 30, 60, and 120 minutes, following the ingestion of glucose. The results from Supplementary Figure 6 indicate that the hyperglycaemic control (Group 6) exhibited impaired glucose tolerance compared to the normal glycaemic (Group 5). However, both the hyperglycaemic control treated with *LB13243* (Group 7) and the hyperglycaemic control treated with metformin (Group 8) groups showed improvements in glucose regulation. These improvements were evidenced by lower blood glucose levels and a more rapid decline after glucose ingestion. This suggests that both the *LB13243* treatment and the control treatment have positive effects on glucose control in the context of diabetes. In another study by Archer et al., the administration of potential probiotic *Lactobacillus fermentum* MCC2759 and MCC2760 improved oral glucose tolerance and insulin levels in rat models fed a high-fat diet and induced with streptozotocin to induce diabetes. The results suggest that these specific *Lactobacillus fermentum* strains may have potential therapeutic benefits for improving glucose metabolism and insulin sensitivity (Archer et al., 2021). Yadav et al. furthermore, a diet supplemented with dahi (a type of yogurt) was found to reduce elevated blood glucose levels, glycosylated hemoglobin, insulin levels, and lipid levels compared to the control group. It also decreased oxidative stress in the liver and pancreas, suggesting that dahi supplementation may help improve glucose regulation and reduce diabetes-related complications (Yadav et al., 2008).

In a study by Srihari et al., the supplementation of kombucha significantly reduced glycosylated hemoglobin levels and increased plasma insulin, hemoglobin, and tissue glycogen levels. This indicates that kombucha supplementation may have a positive impact on glucose control and insulin levels (Srihari et al., 2013). These findings collectively suggest that certain probiotics, kombucha, and specific dietary supplements like dahi may offer potential benefits for individuals with impaired glucose tolerance or diabetes. They have the potential to positively influence glucose control, insulin levels, and related metabolic parameters. However, further research is necessary to fully elucidate the mechanisms behind these effects and to identify the most effective strategies for using probiotics and dietary interventions in the management of diabetes and glucose metabolism disorders.

#### 3.4.3 Serum biochemical analysis

In the antihyperlipidemic study (Supplementary Figure 7A), the group fed an HFD (Group 2) showed higher levels of triglycerides, cholesterol, HDL, LDL, and VLDL compared to the other groups. In the group HFD treated with *LB13243* (Group 3), there were generally lower levels of HDL and LDL compared to the other groups. The HFD-treated rosiglitazone (Group 4) group showed lipid levels similar to the normal group but with some variations in LDL and VLDL levels. A comparison between the normal (Group 1) and *LB13243* groups revealed slightly elevated total cholesterol and VLDL cholesterol levels in the *LB13243* group, while HDL and LDL cholesterol levels were lower. These findings suggest that *LB13243* treatment might have influenced lipid parameters, potentially impacting cholesterol metabolism.

Previous studies have shown that certain *Lactobacillus* strains can regulate lipid metabolism by reducing total cholesterol, triglycerides, and LDL levels while increasing HDL levels in the serum of obese mice (Wang et al., 2020). *Enterococcus* strains have shown potential in reducing cholesterol levels (Ghatani et al., 2022). Xie et al. study involving LAB supplementation demonstrated significant decreases in serum total cholesterol, LDL-C, and triglyceride levels, along with increased HDL-C levels. The LAB-treated groups in this study also had lower hepatic cholesterol and triglyceride levels, reduced liver lipid deposition, and increased fecal cholesterol and bile acid levels (Xie et al., 2011). In our study, the biochemical analysis of serum showed that potential probiotic treatment, particularly *LB13243*, had an impact on lipid parameters, potentially influencing cholesterol metabolism. These findings align with previous studies, indicating that certain probiotics can play a role in regulating lipid levels and promoting a healthier lipid profile.

In the antihyperglycemic study 2 (Supplementary Figure 7B), the results indicate that *LB13243* treatment in the DC + *LB13243* (Group 7) might have potential antihyperglycemic effects. This treatment appears to decrease levels of TG, total cholesterol, LDL cholesterol, and VLDL cholesterol while increasing levels of HDL cholesterol. These findings suggest a potential improvement in lipid profiles and glucose regulation, indicating that the probiotic treatment could positively influence metabolic health. Furthermore, by the end of the experimental period, there was a noticeable normalization of lipid levels in the groups that received potential probiotic bacteria. This normalization suggests that the probiotics may have played a role in balancing lipid parameters and promoting better metabolic outcomes. Supporting these findings, Li et al. conducted a study suggesting that the administration of specific probiotic bacteria, *L. acidophilus* KLDS1.1003 and KLDS1.0901, reduced hepatic triglyceride and total cholesterol levels in male Wistar albino rats. These results indicate that probiotics may have beneficial effects on liver health and lipid metabolism (Li et al., 2021). Similarly, Yan et al. found that *L. acidophilus* treatments led to lower levels of fasting blood glucose (FBG) and glycosylated hemoglobin (HbA1c). This indicates that probiotics may have a positive impact on glycemic control, which is crucial for managing diabetes (You et al., 2020).

The results from both studies indicate that the HFD (Group 2) and DC (Group 6) groups generally show higher values for most biochemical markers compared to the Normal (Group 1) and glycemic control (Group 5) groups, respectively. However, the administration of *LB13243* seems to have a normalizing effect on these markers, as evidenced by the lower values observed in the HFD+*LB13243* (Group 3) and DC+*LB13243* (Group 4) groups compared to the HFD (Group 2) and DC (Group 6) groups, respectively. The results suggest that *LB13243* treatment may have potential benefits in mitigating the negative effects of a high-fat diet and diabetes on the measured biochemical markers. *LB13243* treatment in both anti-hyperlipidaemic and anti-hyperglycaemic studies showed a trend toward normalizing protein metabolism, kidney function, glucose regulation, and liver health (Table 3).

**Table 3:** Serum biochemical changes of anti-hyperlipidaemic and anti-hyperglycaemic study groups.

		Total protein (g/dL)^*^	Uric acid (mg/dL)^*^	Urea (mg/dL)^*^	Creatinine (mg/dL)^*^	Glucose (mg/dL)^*^	Albumin (g/dL)^*^	SGPT (U/L)^*^
Antihyperlipidaemic study	Normal	7.45 ± 0.23^c^	5.12 ± 0.56^b^	24.14 ± 0.84^b^	0.51 ± 0.45^a^	121.27 ± 0.45^b^	3.15 ± 0.14^a^	36.45 ± 0.14^a^
	HFD	7.96 ± 0.46^d^	6.42 ± 0.71^c^	26.14 ±^b^	0.62 ± 0.13^c^	181.12 ± 0.06^c^	4.54 ± 0.52^d^	42.45 ± 0.15^c^
	HFD+*LB13243*	6.25 ± 0.51^a^	4.18 ± 0.36^a^	11.18 ± 0.86^a^	0.53 ± 0.04^a^	101.73 ± 0.74^a^	3.65 ± 0.12^c^	37.45 ± 0.61^b^
	Control	7.85 ± 0.96^b^	5.45 ± 0.89^b^	16.14 ± 0.17^a^	0.59 ± 0.15^b^	111.17 ± 0.82^a^	3.07 ± 0.17^a^	38.51 ± 0.45^b^
Antihyperglycemic study	DC	6.69 ± 0.56^d^	7.56 ± 0.52^d^	46.12 ± 0.95^d^	0.92 ± 0.08^e^	339.22 ± 0.64^e^	3.45 ± 0.23^b^	45.15 ± 0.63^c^
	DC+*LB13243*	6.17 ± 0.91^b^	4.08 ± 0.61^a^	31.18 ± 0.64^c^	0.63 ± 0.35^c^	198.17 ± 0.42^c^	3.39 ± 0.26^b^	39.89 ± 0.11^b^
	Control	6.94 ± 0.26^a^	7.58 ± 0.15^d^	34.42 ± 0.37^c^	0.79 ± 0.53^d^	201.74 ± 0.42^d^	3.51 ± 0.73^b^	39.99 ± 1.15^b^

^*^The result values are expressed as Mean ± SE. DMRT indicates that the means in the same column denoted by different letters (a–d) are significantly distinct (*p* ≤ 0.05).

#### 3.4.4 RNA extraction and qRT-PCR analysis for evaluation of marker genes

Obesity and diabetes are interrelated metabolic disorders characterized by chronic inflammation. Understanding their complex relationship is essential for developing effective prevention and treatment strategies, ultimately enhancing the health and wellbeing of individuals affected by these conditions (Durand et al., 2020). PPAR-γ and C/EBP-α are crucial transcription factors involved in the regulation of gene expressions related to adipogenesis, lipid metabolism, and insulin sensitivity. Their interactions and functions in adipose tissue are critical for maintaining proper metabolic balance, and their dysregulation can lead to metabolic disorders like obesity and diabetes (Musso et al., 2010).

In the antihyperlipidemic study of adipose tissue, PPAR-γ and C/EBP-α gene expressions were consistently upregulated across all treatment groups (Supplementary Figure 8A). This indicates a general increase in these transcription factors with the treatments. Conversely, FAS gene expression was significantly downregulated in the HFD (Group 2) and HFD+*LB13243* (Group 3) groups but upregulated in the Control Rosiglitazone group (Group 4). FABP4 gene expression decreased in both HFD (Group 2) and HFD+*LB13243* (Group 3) groups, with the most notable reduction observed in the Control Rosiglitazone (Group 4). Adiponectin gene expression was reduced in all treated groups (Groups 3 & 4), which might indicate a decrease in adiponectin levels due to the treatments. TNF-α and IL-6 gene expressions were downregulated across all treatment groups (Groups 3 & 4), suggesting an anti-inflammatory effect. GLUT4 gene expression was upregulated in the Normal and HFD+*LB13243* groups (Groups 1 & 3), akin to the Control Rosiglitazone group (Group 4), indicating a potential role for *LB13243* and Rosiglitazone in promoting GLUT4 expression in adipose tissue.

In the antihyperlipidemic study of liver tissue (Supplementary Figure 8B), PPAR-γ and C/EBP-α gene expressions were upregulated in all treatment groups, suggesting their involvement in mediating antihyperlipidemic effects in the liver. FAS gene expression increased in the HFD (Group 2), HFD+*LB13243* (Group 3), and Control Rosiglitazone groups (Group 4), potentially affecting fatty acid synthesis. FABP4 gene expression was elevated in the HFD (Group 2), and HFD+*LB13243* (Group 3) groups but decreased in the Control Rosiglitazone (Group 4) group. Adiponectin gene expression was upregulated in the HFD+*LB13243* (Group 3) and Control Rosiglitazone groups (Group 4), which may enhance insulin sensitivity and exhibit anti-inflammatory effects. TNF-α gene expression was consistently downregulated in all treated groups, indicating an inhibitory effect. IL-6 gene expression was upregulated in the HFD (Group 2), and HFD+*LB13243* (Group 3) groups but reduced in the Control Rosiglitazone group (Group 4), demonstrating differential regulation of IL-6 expression.

These results provide significant insights into the regulatory effects of treatments on genes involved in lipid metabolism, inflammation, and glucose homeostasis in adipose and liver tissues. They emphasize the necessity for further research to confirm these findings and explore their therapeutic potential. For instance, Zhu et al. identified *Lactobacillus fermentum* LF-CQPC05 from Sichuan pickles and observed its influence on lipid metabolism regulators such as lipoprotein lipase and PPAR-α. This strain could potentially aid in managing lipid-related disorders (Zhu et al., 2019).

Similarly, Park et al. (2019) proposed *Lactobacillus amylovorus* KU4 as a potential obesity therapeutic by modulating PPAR-γ signaling, while Long et al. (2020) highlighted *Lactobacillus* KFY04′s ability to mitigate oxidative damage and inflammation through the PPAR pathway.

Mu et al. (2020) demonstrated that *Lactobacillus plantarum*-KFY02 upregulates key lipid metabolism genes while downregulating adipogenic factors. Voltan et al. showed that *Lactobacillus crispatus* M247 enhances PPAR-γ activity in the colonic mucosa, which could impact gut health and inflammation. This study adds to the growing body of evidence supporting the beneficial effects of *Lactobacillus* strains in modulating lipid and glucose metabolism (Voltan et al., 2008).

In the antihyperglycemic study, gene expression analysis revealed that PPAR-γ and C/EBP-α were upregulated in the Liver, Pancreas, and Adipose tissues across all treated groups, including *LB13243* and metformin (Supplementary Figure 9). This indicates that both treatments (Group 7&8)may modulate these genes, influencing lipid metabolism and adipocyte differentiation. FAS gene expression was notably downregulated in Liver and Adipose tissues in the DC (group 6) and DC+*LB13243* (group 7) groups, suggesting suppression of fatty acid synthesis. FABP4 expression also decreased in Liver and Pancreas tissues in these groups (groups 7&8). Conversely, Adiponectin gene expression increased in Liver and Adipose tissues, highlighting its role in insulin sensitivity and anti-inflammatory effects. The consistent downregulation of TNF-α and IL-6 gene expressions in all treated tissues implies potential anti-inflammatory benefits. GLUT4 gene expression was significantly upregulated in all treated tissues, suggesting enhanced glucose uptake.

Hsieh et al. (2013) reported that *Lactobacillus reuteri* GMNL-263 supplementation improved gut microbiota balance and enhanced antioxidant enzyme activity, leading to increased PPAR-γ and GLUT4 expressions. Phenylacetic acid (PLA) from *Lactobacillus plantarum* was found to promote adipocyte differentiation and glucose uptake (Ilavenil et al., 2015). Additionally, *Lactobacillus plantarum* NCU116 upregulated key regulators involved in lipid and glucose metabolism (Li et al., 2014).

These findings suggest that *L. plantarum* NCU116 may have potential implications in improving lipid and glucose metabolism, which are critical factors in metabolic health. PPAR-γ and C/EBP-a were consistently upregulated in all treated tissues, potentially impacting lipid metabolism and adipocyte differentiation (Mu et al., 2020). FAS and FABP4 were downregulated in Liver and Adipose tissues, suggesting suppression of fatty acid synthesis and transport (Wu et al., 2020). Adiponectin was upregulated in Liver and Adipose tissues, known for insulin-sensitizing and anti-inflammatory effects. TNF-a and IL-6 were downregulated, indicating inhibition of pro-inflammatory genes (Archer et al., 2021). GLUT4 was upregulated, potentially influencing glucose uptake and metabolism in all treated tissues (Gandhi et al., 2014). These findings highlight the potential antihyperglycemic effects and offer valuable insights into the molecular mechanisms underlying the treatments. Our study findings underscore the potential therapeutic effects of *LB13243* and Rosiglitazone in antihyperlipidemic conditions and *LB13243* and metformin in antihyperglycemic conditions. They contribute to a deeper understanding of the molecular mechanisms involved and offer insights into potential strategies for targeting lipid and glucose metabolism.

#### 3.4.5 Tissue processing and histopathological studies

**Figure 4:**
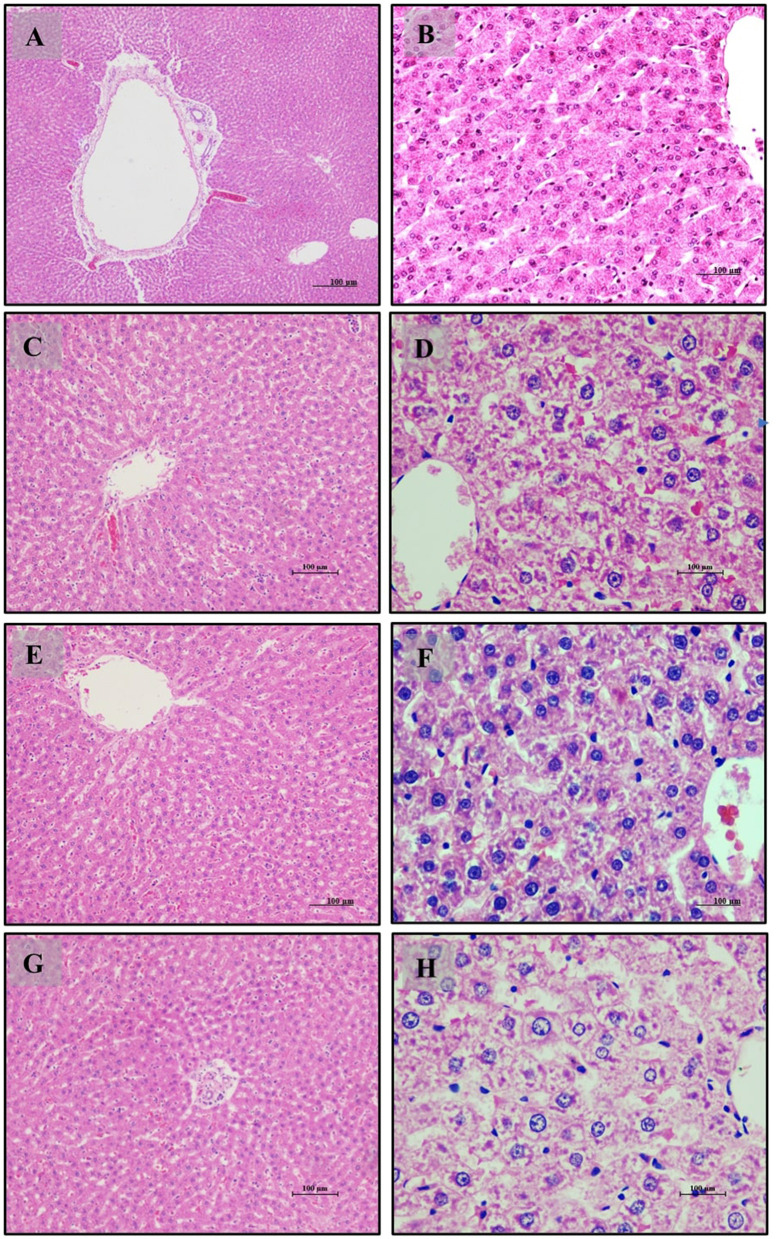
Liver of male Wistar albino rat. Antihyperlipidemic study 1. H and E, ×20 and ×40: **(A, B)** healthy diet normal control group; **(C, D)** high fat-fed diet control group; **(E, F)** high fat-fed diet group treated with *LB13243* (10^8^ CFU/mL); and **(G, H)** high fat fed group given positive control drug Rosiglitazone (0.7 mg/Kg of body weight).

In this antihyperlipidemic study, we examined the histopathological changes in Wistar albino rats subjected to various dietary interventions. Tissues were processed using hematoxylin and eosin (H&E) staining and analyzed under ×20 and ×40 magnifications, as illustrated in Figure 4.

Liver tissue analysis revealed that rats on a normal diet (Group 1) exhibited intact hepatic parenchyma with well-preserved portal triads and hepatocyte organization, as seen in Figures 4A, B. Hepatocytes were organized in regular hexagonal lobules with uniformly distributed nuclei, and no fat globules were present. Conversely, the high-fat diet group (Group 2) displayed significant disturbances in hepatic architecture, including the presence of small fat globules throughout the hepatic cytoplasm and evident hepatocyte disorganization. Prominent steatosis with varying sizes of lipid droplet vacuoles was observed in Figures 4C, D. Treatment with *LB13243* in the high-fat diet group (Group 3) led to near-normal liver lobular structure, with regular hexagonal shapes and similarly sized nuclei, and a notable reduction in intracellular fat globules, restoring hepatic architecture to a condition comparable to the normal diet group, as shown in Figures 4E, F. The positive control group treated with Rosiglitazone (Group 4) demonstrated improvements in hepatic structure, including a reduction in fat globules and some evidence of nuclear dissolution, indicating partial restoration of liver architecture (Figures 4G, H).

**Figure 5:**
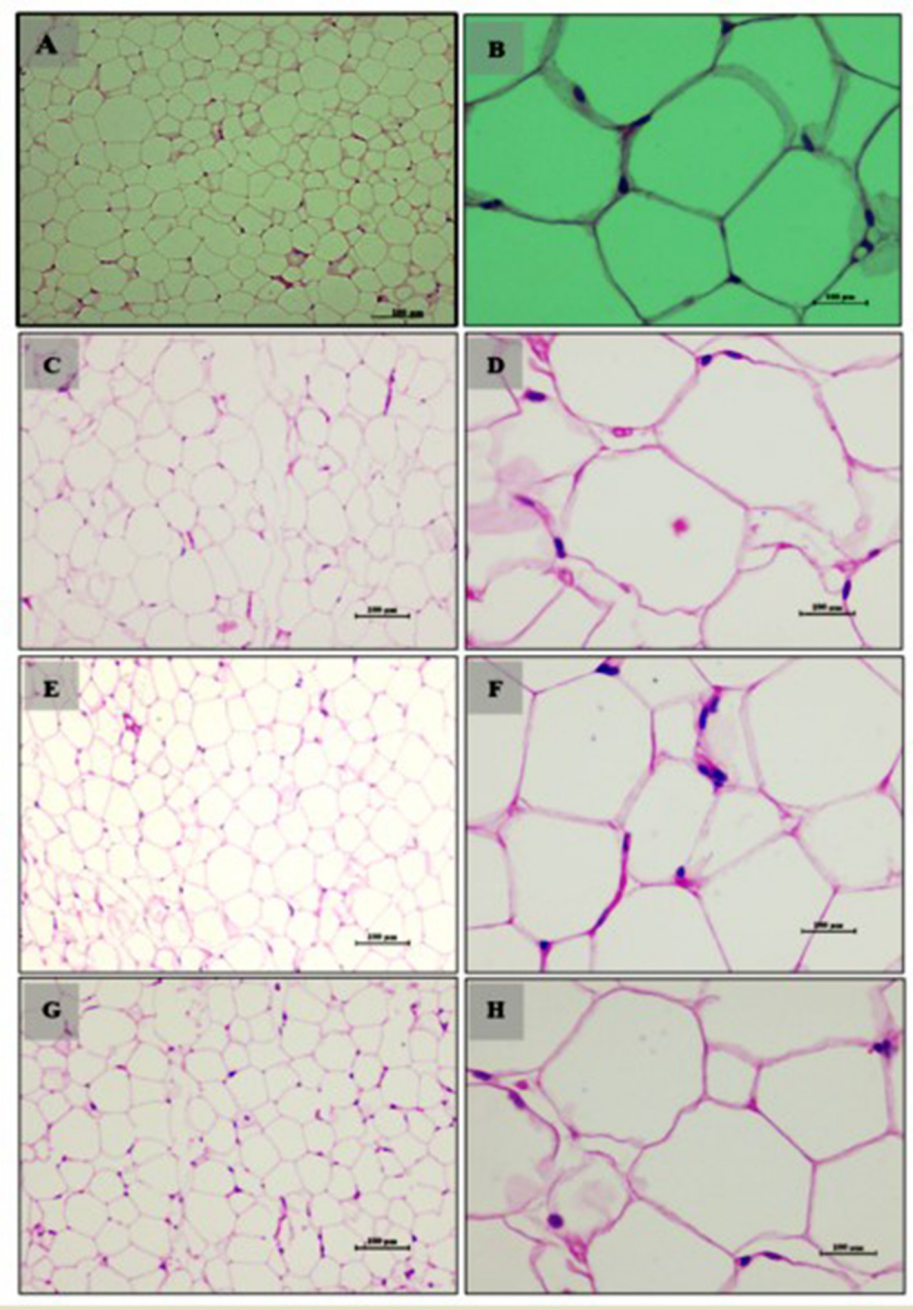
Adipocytes (Retroperitoneal adipose tissue-visceral fat) of male Wistar albino rat. Antihyperlipidemic activity study 1. H and E, ×20 and ×40: **(A, B)** healthy diet normal control group; **(C, D)** high fat-fed diet control group; **(E, F)** high fat-fed diet group treated with *LB13243* (10^8^ CFU/mL); and **(G, H)** high fat fed group given positive control drug Rosiglitazone (0.7 mg/Kg of body weight).

Adipose tissue analysis showed that rats on a normal diet (Group 1) had well-differentiated adipocytes with no observable abnormalities (Figures 5A, B). In contrast, the high-fat diet group (Group 2) exhibited increased adipocyte size and number, indicative of lipid accumulation, hypertrophy, and hyperplasia (Figures 5C, D). The addition of *LB13243* to the high-fat diet (Group 3) led to reduced adipocyte size and number, decreased lipid accumulation, and improved overall tissue health (Figures 5E, F). Similarly, the positive control group treated with Rosiglitazone (Group 4) showed improvements in adipose tissue, including reduced adipocyte size and number (Figures 5G, H).

**Figure 6:**
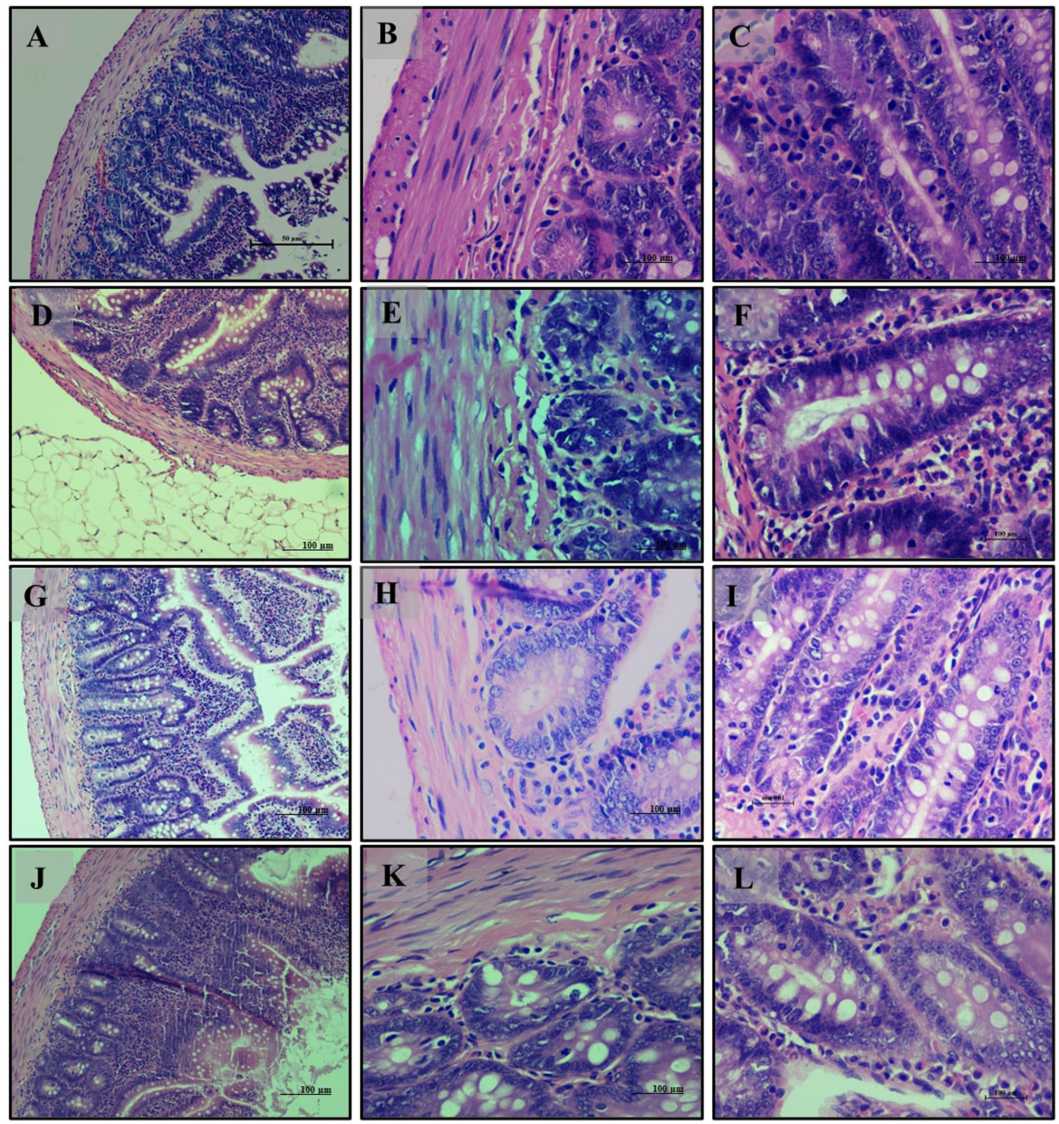
Small Intestine of male Wistar albino rat. Antihyperlipidemic activity study 1. H and E, ×20 and ×40: **(A–C)** healthy diet normal control group; **(D–F)** high fat-fed diet control group; **(G–I)** high fat-fed diet group treated with *LB13243* (10^8^ CFU/mL); and **(J–L)** high fat fed group given positive control drug Rosiglitazone (0.7 mg/Kg of body weight).

Small intestine analysis revealed a normal histological appearance with intact villi and a healthy mucosal lining in the normal diet group (Group 1) (Figures 6A–C). The high-fat diet group (Group 2) showed disruptions in tissue structure, characterized by disorganized villi and lipid accumulation in epithelial cells (Figures 6D–F). Treatment with *LB13243* (Group 3) improved tissue structure and reduced lipid accumulation, though some fat infiltration persisted (Figures 6G–I). The positive control group (Group 4) also demonstrated moderate structural improvements and reduced fat accumulation, with some persistent fat globules (Figures 6J–L).

**Figure 7:**
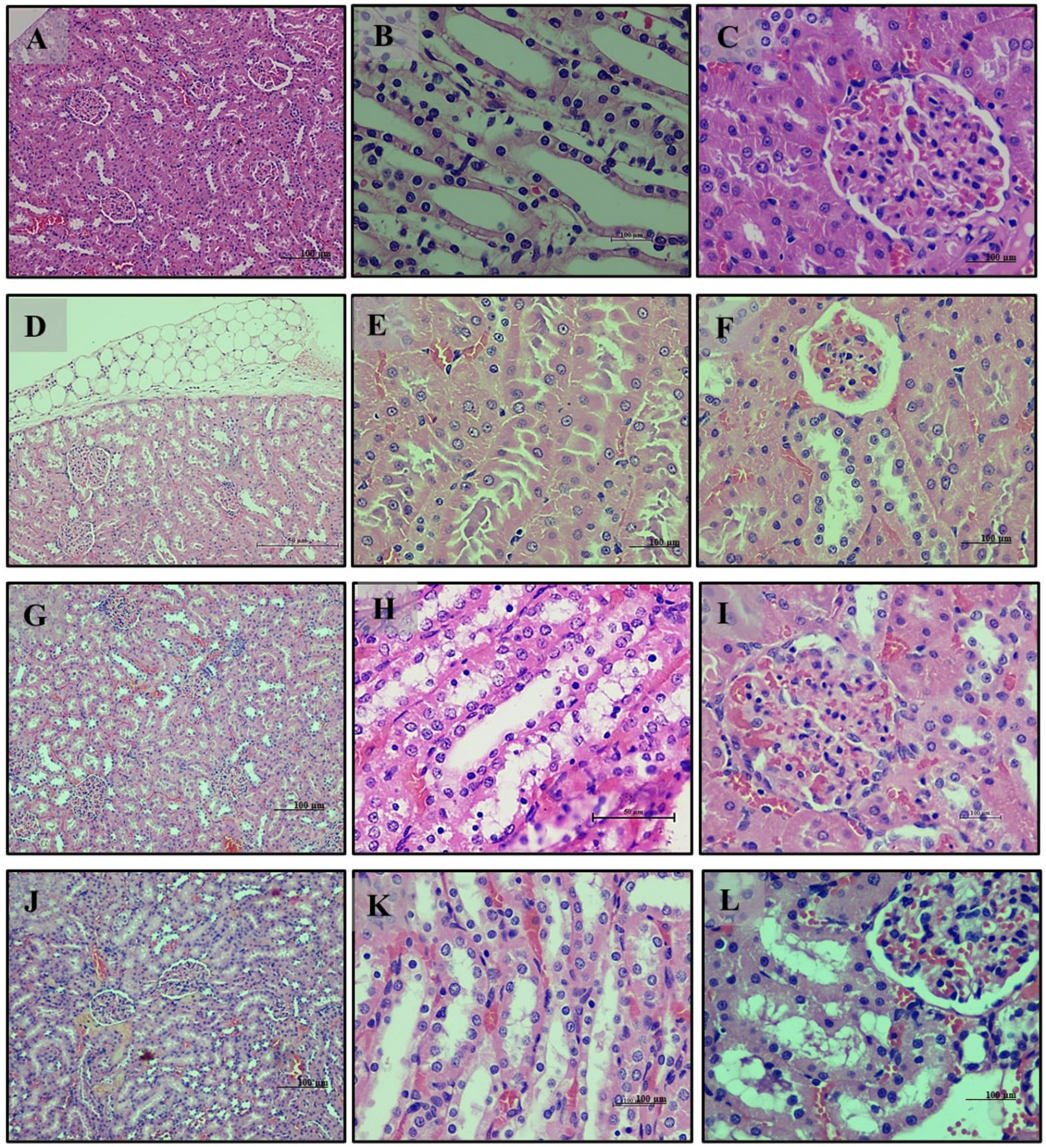
Kidney of male Wistar albino rat. Antihyperlipidemic activity study 1. H and E, ×20 and ×40: **(A–C)** healthy diet normal control group; **(D–F)** high fat-fed diet control group; **(G–I)** high fat-fed diet group treated with *LB13243* (10^8^ CFU/mL); and **(J–L)** high fat fed group given positive control drug Rosiglitazone (0.7 mg/Kg of body weight).

Kidney analysis revealed normal renal histology with well-differentiated tubules in the normal diet group (Group 1) (Figures 7A–C). The high-fat diet group (Group 2) exhibited disrupted renal tissue structure, increased lipid accumulation, and vascular engorgement (Figures 7D–F). The addition of *LB13243* (Group 3) led to improvements in kidney structure, with reduced lipid accumulation and a more regular tubule arrangement (Figures 7G–I). The positive control group (Group 4) also showed improvements, including reduced lipid accumulation, although some glomerular architectural disruptions remained (Figures 7J–L).

Our antihyperlipidemic study highlights that a high-fat diet induces significant histopathological changes across multiple tissues, characterized by increased lipid accumulation and structural disruptions. Both *LB13243* and Rosiglitazone treatments demonstrate promising effects in mitigating these adverse changes and improving tissue health.

**Figure 8:**
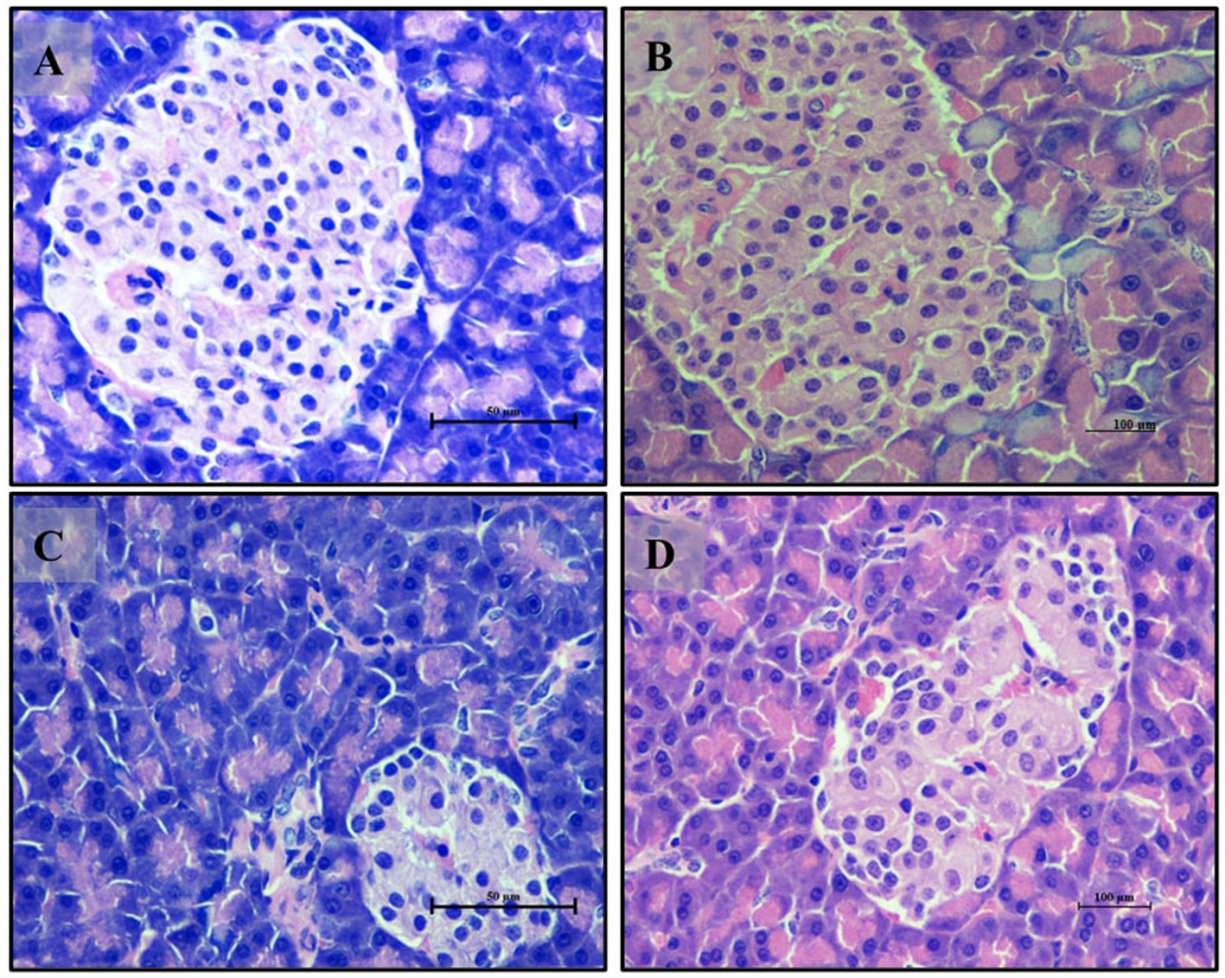
Pancreas of male Wistar albino rat. Antihyperglycemic activity study 2. H and E, ×40: **(A)** normal glycaemic control group; **(B)** hyperglycaemic control group; **(C)** hyperglycaemic group treated with *LB13243* (10^8^ CFU/mL); and **(D)** hyperglycaemic group treated with positive control drug Metformin (0.1 mg/Kg of body weight).

The histopathology study of antihyperglycemic study for pancreas sections from the normal glycemic control group (Group 6) (Figure 8A) displayed a healthy histological appearance with well-preserved pancreatic tissue. The islets of Langerhans were intact and organized, and the acinar cells showed a normal arrangement. In contrast, the hyperglycemic control group (Group 7) (Figure 8B) exhibited significant histological disruptions, including irregular and disorganized islets of Langerhans and alterations in the acinar cells, indicative of potential functional impairment. The group treated with *LB13243* (Group 8) (Figure 8C) demonstrated notable improvements, with tissue structure resembling that of the normal glycemic control group, indicating potential antihyperglycemic effects. Similarly, the group treated with Metformin (Group 9) (Figure 8D) also showed improved tissue morphology, with better-preserved islets and acinar cells compared to the hyperglycemic control group.

**Figure 9:**
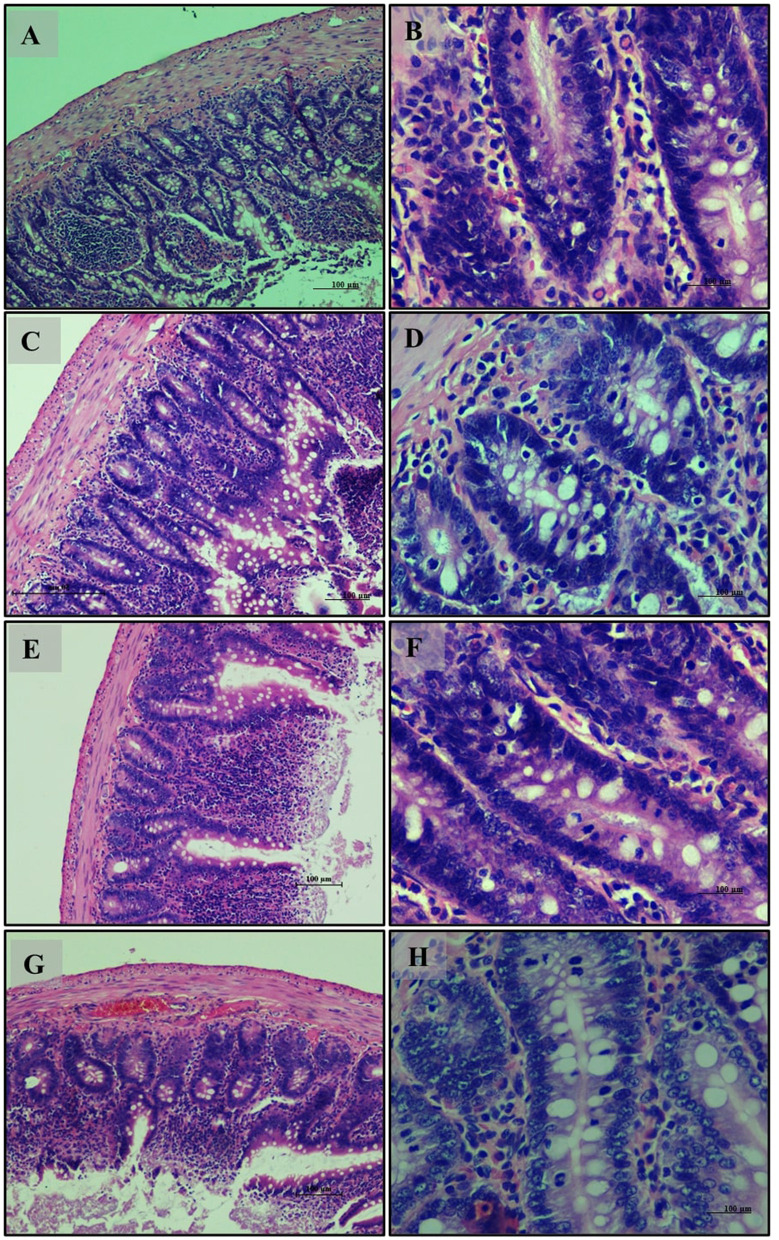
Small Intestine of male Wistar albino rat. Antihyperglycemic activity study 2: H and E, ×20 and ×40: **(A, B)** normal glycaemic control group; **(C, D)** hyperglycaemic control group; **(E, F)** hyperglycaemic group treated with *LB13243* (10^8^ CFU/mL); and **(G, H)** hyperglycaemic group treated with positive control drug Metformin (0.1 mg/Kg of body weight).

Small intestine sections from the normal glycemic control group (Group 6) (Figures 9A, B) showed a well-organized intestinal structure with healthy villi and mucosal lining, and an adequate presence of mucus-producing goblet cells. The hyperglycemic control group (Group 7) (Figures 9C, D) exhibited disruptions in intestinal structure, including irregular and disorganized villi and a reduced presence of goblet cells, suggesting impaired intestinal function. In the group treated with *LB13243* (Group 8) (Figures 9E, F), the tissue structure improved, resembling that of the normal glycemic control group with well-organized villi and enhanced goblet cell presence. The Metformin-treated group (Group 9) (Figures 9G, H) also showed improvements in tissue structure, with organized villi and better-preserved goblet cells.

**Figure 10:**
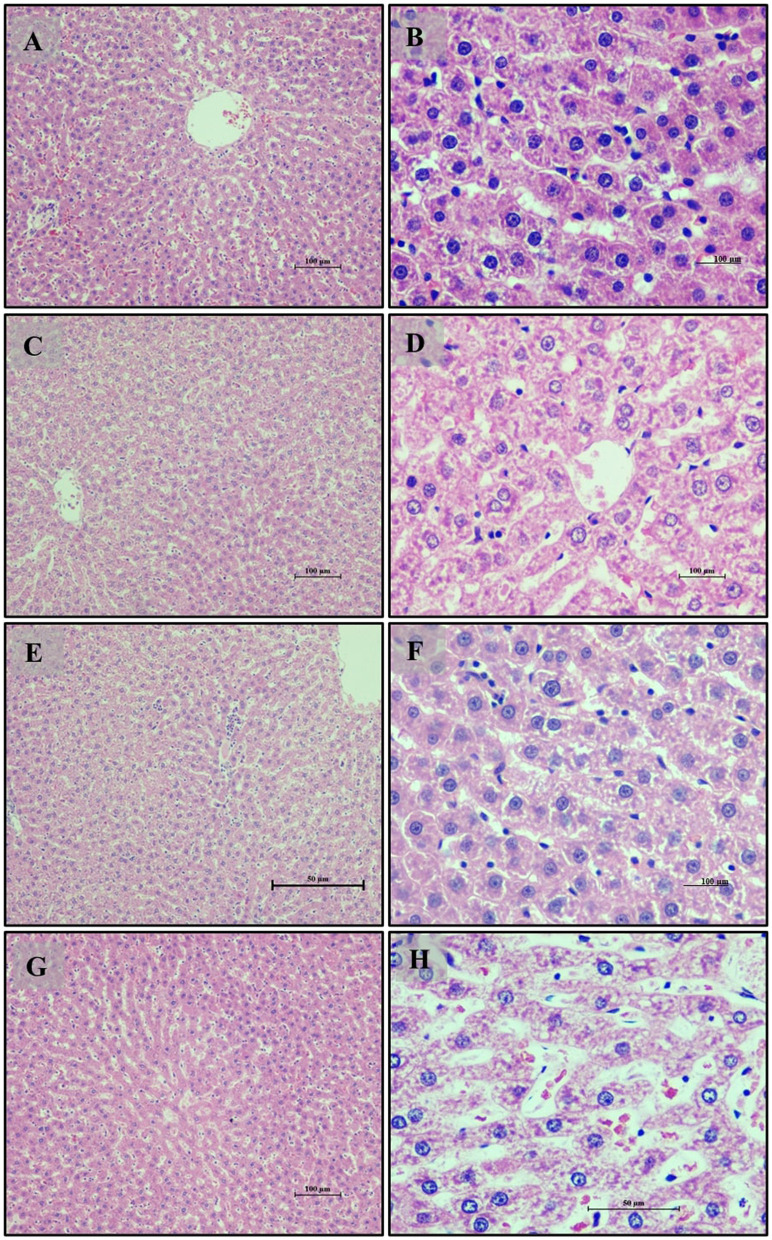
Liver of male Wistar albino rat. Antihyperglycemic activity study 2: H and E, ×20 and ×40: **(A, B)** normal glycaemic control group; **(C, D)** hyperglycaemic control group; **(E, F)** hyperglycaemic group treated with *LB13243* (10^8^ CFU/mL); and **(G, H)** hyperglycaemic group treated with positive control drug Metformin (0.1 mg/Kg of body weight).

The liver sections from the normal glycemic control group (Group 6) (Figures 10A, B) exhibited healthy liver architecture with well-differentiated hepatocytes and regular hepatic lobules. In contrast, the hyperglycemic control group (Group 7) (Figures 10C, D) showed notable disruptions, including disorganized hepatocyte arrangement, increased cell size (hypertrophy), and distorted hepatic sinusoids. The liver sections from the *LB13243*-treated group (Group 8) (Figures 10E, F) demonstrated marked improvements, with a more regular hepatocyte arrangement and reduced hypertrophy, indicating potential antihyperglycemic effects. Similarly, the Metformin-treated group (Group 9) (Figures 10G, H) showed improvements with better hepatocyte organization and reduced hypertrophy compared to the hyperglycemic control group.

**Figure 11:**
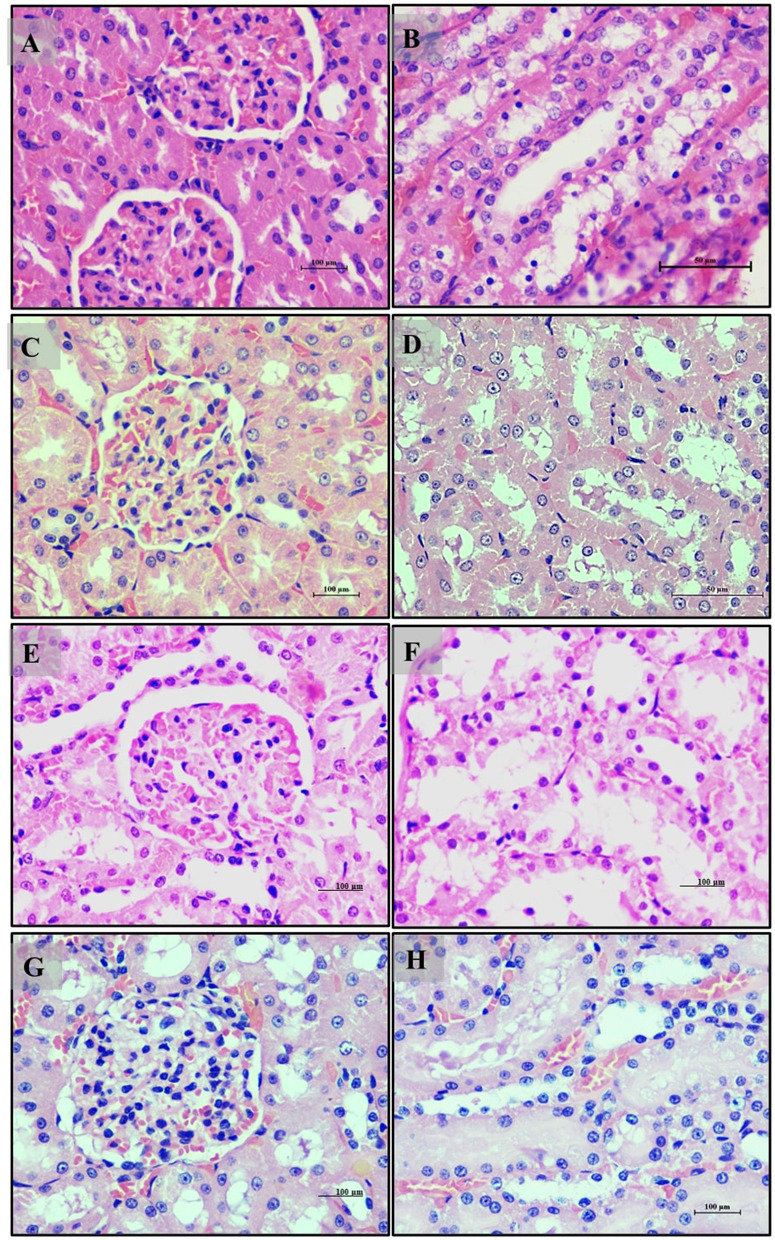
Kidney of male Wistar albino rat. Antihyperglycemic activity study 2: H and E, ×20 and ×40: **(A, B)** normal glycaemic control group; **(C, D)** hyperglycaemic control group; **(E, F)** hyperglycaemic group treated with *LB13243* (10^8^ CFU/mL); and **(G, H)** hyperglycaemic group treated with positive control drug Metformin (0.1 mg/Kg of body weight).

Kidney sections from the normal glycemic control group (Group 6) (Figures 11A, B) displayed a normal histological appearance with well-preserved glomeruli and renal tubules. The hyperglycemic control group (Group 7) (Figures 11C, D) exhibited degeneration of glomeruli and renal tubules, loss of nuclei, and signs of fibrosis in the interstitial spaces, indicating potential renal damage. The group treated with *LB13243* (Group 8) (Figures 11E, F) showed improvements, with more regular glomeruli and renal tubules and reduced interstitial fibrosis, suggesting potential antihyperglycemic effects. The Metformin-treated group (Group 9) (Figures 11G, H) also displayed improvements, though with some residual signs of tubular degeneration and interstitial damage, less pronounced than in the hyperglycemic control group.

**Figure 12:**
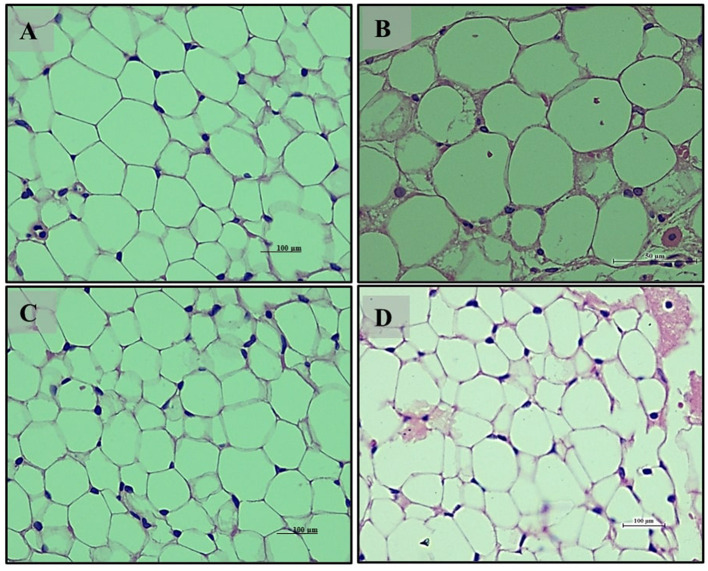
Adipocytes (Retroperitoneal adipose tissue-visceral fat) of male Wistar albino rat. Antihyperglycemic activity study 2. H and E, ×40: **(A)** normal glycaemic control group; **(B)** hyperglycaemic control group; **(C)** hyperglycaemic group treated with *LB13243* (10^8^ CFU/mL); and **(D)** hyperglycaemic group treated with positive control drug Metformin (0. mg/Kg of body weight).

Adipocyte section from the normal glycemic control group (Group 6) (Figure 12A) exhibited well-differentiated adipocytes with regular size and arrangement. The hyperglycemic control group (Group 7) (Figure 12B) showed disrupted adipose tissue structure, with enlarged and irregularly arranged adipocytes, reflecting altered metabolic function due to hyperglycemia. The *LB13243*-treated group (Group 8) (Figure 12C) demonstrated improvements, with adipocytes resembling those of the normal glycemic control group in size and arrangement. Similarly, the Metformin-treated group (Group 9) (Figure 12D) also displayed improvements, with adipocytes appearing more regular and similar to the normal glycemic control group. Overall, in our antihyperglycemic study, Metformin demonstrated potential antihyperglycemic effects by improving histological outcomes in pancreatic, intestinal, liver, kidney, and adipose tissues affected by hyperglycemia.

## 4 Summary and conclusion

The probiotic strains isolated from fermented sugarcane juice, particularly *Levilactobacillus brevis* RAMULAB54, a novel approach that provides evidence for the potential therapeutic effects of *LB13243* in the management of hyperlipidemia and hyperglycemia. This research offers a comprehensive evaluation of LB13243 and *Levilactobacillus brevis* RAMULAB54 for managing metabolic disorders through a robust, multi-faceted approach. The molecular docking and dynamics simulations confirmed LB13243′s stable interactions with target proteins and favorable pharmacokinetic properties, underscoring its therapeutic potential. Cellular assays demonstrated *LB13243*′*s* low cytotoxicity and its significant impact on adipocyte differentiation, which is crucial for understanding its role in metabolic regulation. *In vivo* studies highlighted its positive effects on food intake, body weight, organ weights, glucose tolerance, and serum biochemical profiles, illustrating its beneficial influence on overall metabolic health. Histopathological analyses reinforced these findings by showing restored tissue architecture and reduced lipid accumulation. The significance of this study lies in its potential to advance the development of new therapeutic strategies for dyslipidemia and hyperglycemia, addressing significant health concerns associated with metabolic disorders. By providing a detailed assessment of *LB13243* and *Levilactobacillus brevis* RAMULAB54, this research contributes valuable insights into their efficacy and supports their potential for further development and clinical evaluation.

In conclusion, this work presents a thorough characterization of LAB isolates from fermented foods, demonstrating their broad species composition and emphasizing their capacity to survive in particular environmental circumstances. Gene expression and histopathological analyses further supported the beneficial effects of *L. brevis* on metabolic health, indicating its potential as a therapeutic agent for obesity and obesity-associated diabetes. Overall, this study enhances our understanding of LAB diversity, probiotic properties, and the therapeutic potential of *L. brevis* in metabolic conditions. These results lay the foundation for further experimental investigations and the development of peptide-based therapeutics or functional foods with specific health-promoting effects in managing metabolic conditions.

## Data Availability

The data presented in this study are deposited in the NCBI GenBank repository, accession number ON872226.1.
