# Supplementary material for: Fermented sugarcane juice-derived probiotic Levilactobacillus brevis RAMULAB54 enhances lipid metabolism and glucose homeostasis through PPAR-γ activation
Source: Front Microbiol. 2025 Jan 29;15:1502751. doi: 10.3389/fmicb.2024.1502751 (PMC11814229; doi:10.3389/fmicb.2024.1502751)
Supplement: Supplementary file 1 [file Data_Sheet_1.docx]

**Research Paper**

**Fermented sugarcane juice-derived probiotic *Levilactobacillus brevis* RAMULAB54 Enhances Lipid Metabolism and Glucose Homeostasis through PPAR-γ Activation**

**Chandana Kumari V B ^1†^, Ramith Ramu ^1†^*, Sujay S Huligere ^1^, Shashank M Patil ^1^, Shivasharanappa Nayakvadi ^2^, Sharath Bijoor ^3^, Uma Venkateswaran Manjappara ^4^, Mohammad Z. Ahmed ^5^, Ling Shing Wong ^6^**

**^1^** Department of Biotechnology and Bioinformatics, JSS Academy of Higher Education and Research, Mysore – 570015, Karnataka, INDIA [chandanavb2@gmail.com](mailto:chandanavb2@gmail.com) (C.K.V.B.); [sujayhuligere@gmail.com](mailto:sujayhuligere@gmail.com) (S.S.); [shashankmpatil@jssuni.edu.in](mailto:shashankmpatil@jssuni.edu.in) (S.M.P.)

**^2^** ICAR-National Institute of Veterinary Epidemiology and Disease Informatics (NIVEDI), Yelahanka, Bengaluru, Karnataka, India [drshivasharan@gmail.com](mailto:drshivasharan@gmail.com) (S.N.)

**^3^** Department of Plant Cell Biotechnology, CSIR-Central Food Technological Research Institute (CFTRI), Mysore 570-020, India [sharathbijoor@gmail.com](mailto:sharathbijoor@gmail.com) (S.B.)

**^4^** Department of Biochemistry, CSIR-Central Food Technological Research Institute (CFTRI), Mysore 570-020, India [umamanjappara@cftri.res.in](mailto:umamanjappara@cftri.res.in) (U.V.M.)

**^5^** Department of Pharmacognosy, College of Pharmacy, King Saud University, Riyadh 11451, Saudi Arabia [mahmed4@ksu.edu.sa](mailto:mahmed4@ksu.edu.sa) (M.Z.A.)

**^6^** Faculty of Health and Life Sciences, INTI International University, Nilai, Malaysia lingshing.wong@newinti.edu.my (L.S.W.)

**^†^** Equal contribution

*Correspondence: [ramith.gowda@gmail.com](mailto:ramith.gowda@gmail.com)

**Supplementary** **Table 1**: qRT-PCR oligonucleotide sequences used in gene expression analysis.

| **Gene** | **Accession id** | **Primer** | **Sequence (5' to 3')** | **Annealing temperature (°C)** |
| --- | --- | --- | --- | --- |
| **PPAR-γ** | NM_001127330 | F | GTGCTCCAGAAGATGACAGAC | 60 |
|  |  | R | GGTGGGACTTTCCTGCTAA |  |
| **C/EBP-α** | NM_001287521 | F | GTGGACAAGAACAGCAACGA | 59 |
|  |  | R | TTGACCAAGGAGCTCTCAGG |  |
| **FAS** | NM_007988.3 | F | CAAGTGTCCACCAACAAGC | 59 |
|  |  | R | GGAGCGCAGGATAGACTCAC |  |
| **Adiponectin** | NM_009605 | F | GGGTGAGACAGGAGATGTTGGAATG | 60 |
|  |  | R | GCCAGTAAATGTAGAGTCGTTGACG |  |
| **GLUT-4** | NM_001359114 | F | GGAACACTGGTCCTAGCTGT | 59 |
|  |  | R | ATCATGCCACCCACAGAGAA |  |
| **β-actin** | NM_007393 | F | CTCTATGCCAACACAGTGC | 60 |
|  |  | R | CTCAGTAACAGTCCGCCTA |  |

**Supplementary** **Table 2:** Primer used in the gene expression analysis through qRT PCR.

| **Gene Accession no.** | **Primer** | **Sequence (5' to 3')** | **Annealing temperature (°C)** |
| --- | --- | --- | --- |
| **PPAR-γ** | F | GTGCTCCAGAAGATGACAGAC | 60 |
| **XM_032905882.1** | R | GGTGGGACTTTCCTGCTAA |  |
| **C/EBP-α** | F | TTCCTTGGTGAGTTCGTGGA | 59 |
| **NM_001287579.1** | R | GGATGGTCCCAGTGTCTTCA |  |
| **FAS** | F | CCGAGGAACAAACACTGGTG | 59 |
| **X62888.1** | R | GCAATGCTGGGTCCTTTGAA |  |
| **Adiponectin** | F | AAGGTGGTCTTCGGGATGTT | 60 |
| **NM_207587.2** | R | AATAGAGCCAGGGGACGAAG |  |
| **FABP4** | F | ATGTGCAGAAGTGGGATGGA | 55 |
| **NM_053365.3** | R | TGCAAATTTCAGTCCAG |  |
| **TNF-α** | F | CCACCACGCTCTTCTGTCTA | 62 |
| **L19123.1** | R | CCCCAACTCTCCTCCACATT |  |
| **Insulin (INS)** | F | GTGGGGAACGTGGTTTCTTC | 64 |
| **NM_019129.3** | R | TGCAGCACTGATCCACAATG |  |
| **GLUT-4** | F | TCTCTGTGGGTGGCATGATT | 59 |
| **NM_012751.1** | R | AGGCACCAACCCTGATGTTA |  |
| **Insulin Receptor (INSR)** | F | TCTGAGAAGGGGCATCTGTG | 60 |
| **AF005777.1** | R | GGTGGAGGAGATGTTGGGAA |  |
| **IL-6** | F | CCACTGCCTTCCCTACTTCA | 61 |
| **NM_012589.2** | R | TTCTGACAGTGCATCATCGC |  |
| **Glucose-6-Phosphatase (G6PC)** | F | GCTGTTTGAGGAAAGCGTGA | 62 |
| **BC090067.1** | R | CCTCCCGATCCTTCCTTCTC |  |
| **β-actin** | F | CTCTATGCCAACACAGTGC | 60 |
| **NM_031144.3** | R | CTCAGTAACAGTCCGCCTA |  |

**Supplementary** **Table 3**. Virtual screening of organic acids from LBR54 docked to PPARγ protein.

| Organic acids | Binding affinity (kcal/mol) | Total no. of intermolecular bonds | Total no. of hydrogen bonds |
| --- | --- | --- | --- |
| Citric acid | -9.2 | 8 | 2 |
| Fumaric acid | -10.5 | 5 | 3 |
| Hydroxycitric acid | **-11.0** | **7** | **7** |
| Lactic acid | -10.0 | 6 | 4 |
| Maleic acid | -9.8 | 5 | 4 |
| Malic acid | -10.2 | 7 | 5 |
| Malonic acid | -10.6 | 6 | 4 |
| Pyruvic acid | -9.6 | 8 | 5 |
| Shikimic acid | -10.6 | 7 | 4 |
| Succinic acid | -10.8 | 6 | 5 |
| Tartaric acid | -8.6 | 5 | 2 |
| Rosiglitazone | **-12.0** | **12** | **8** |

**Supplementary** **Table 4**. Pharmacokinetic analysis of rosiglitazone and hydroxycitric acid

| Particulars | Hydroxycitric acid | Rosiglitazone |
| --- | --- | --- |
| Molecular weight | 208.12 g/mol | 357.43 g/mol |
| Num. rotatable bonds | 5 | 7 |
| Num. H-bond acceptors | 8 | 4 |
| Num. H-bond donors | 5 | 1 |
| TPSA | 152.36 Å² | 96.83 Å² |
| Consensus Log P | -2.08 | 2.36 |
| GI absorption | Moderate | High |
| BBB permeant | No | No |
| P-gp substrate | No | No |
| CYP1A2 inhibitor | No | Yes |
| CYP2C19 inhibitor | No | Yes |
| CYP2C9 inhibitor | No | Yes |
| CYP2D6 inhibitor | No | Yes |
| CYP3A4 inhibitor | No | Yes |
| DILI | No | Yes |
| AMES Toxicity | No | Yes |

**Supplementary** **Table 5**: Weight (g) of different organs of anti-hyperlipidaemic and anti-hyperglycaemic study groups post necropsy

| Groups | Heart | Liver | Pancreas | Spleen | Adrenal glands | Kidneys | Intestine |
| --- | --- | --- | --- | --- | --- | --- | --- |
| Antihyperlipidemic study | | | | | | | |
| 1 | 0.72 ± 0.01^a^ | 7.12 ± 0.19^a^ | 0.006 ± 0.12^a^ | 0.71 ± 0.19^c^ | 0.036 ± 0.01^a^ | 1.42 ± 0.11^a^ | 5.71 ± 0.22^c^ |
| 2 | 1.53 ± 0.98^d^ | 11.41 ± 1.52^d^ | 0.01 ± 0.15^d^ | 0.85 ± 0.05^d^ | 0.058 ± 0.02^d^ | 2.19 ± 1.23^d^ | 8.15 ± 0.12^d^ |
| 3 | 0.76 ± 0.54^b^ | 8.85 ± 0.06^b^ | 0.008 ± 0.64^b^ | 0.66 ± 0.34^b^ | 0.045 ± 0.02^b^ | 1.72 ± 0.43^b^ | 5.46 ± 0.11^b^ |
| 4 | 0.78 ± 0.01^c^ | 9.13 ± 0.49^c^ | 0.009 ± 0.01^c^ | 0.62 ± 0.53^a^ | 0.047 ± 0.05^c^ | 1.97 ± 0.34^c^ | 5.33 ± 0.25^a^ |
| Antihyperglycemic study | | | | | | | |
| 5 | 0.74 ± 0.01^d^ | 8.12 ± 0.54^a^ | 0.007 ± 0.24^a^ | 0.54 ± 0.16^c^ | 0.031 ± 0.21^a^ | 1.45 ± 0.14^c^ | 5.79 ± 0.01^c^ |
| 6 | 0.65 ± 0.45^c^ | 10.16 ± 0.07^d^ | 0.012 ± 0.11^d^ | 0.79 ± 0.14^d^ | 0.065 ± 0.05^d^ | 1.97 ± 0.05^d^ | 6.89 ± 0.64^d^ |
| 7 | 0.63 ± 0.35^b^ | 9.02 ± 0.82^b^ | 0.009 ± 0.21^b^ | 0.46 ± 0.18^a^ | 0.048 ± 0.01^c^ | 1.23 ± 0.12^b^ | 5.14 ± 1.34^a^ |
| 8 | 0.59 ± 0.15^a^ | 9.72± 1.25^c^ | 0.01 ± 0.15^c^ | 0.48 ± 0.56^b^ | 0.047 ± 0.02^b^ | 1.03 ± 0.21^a^ | 5.18 ± 1.11^b^ |

* The result values are expressed as Mean ± SE. DMRT indicates that the means in the same column denoted by different letters (a–d) are significantly distinct (p ≤ 0.05).


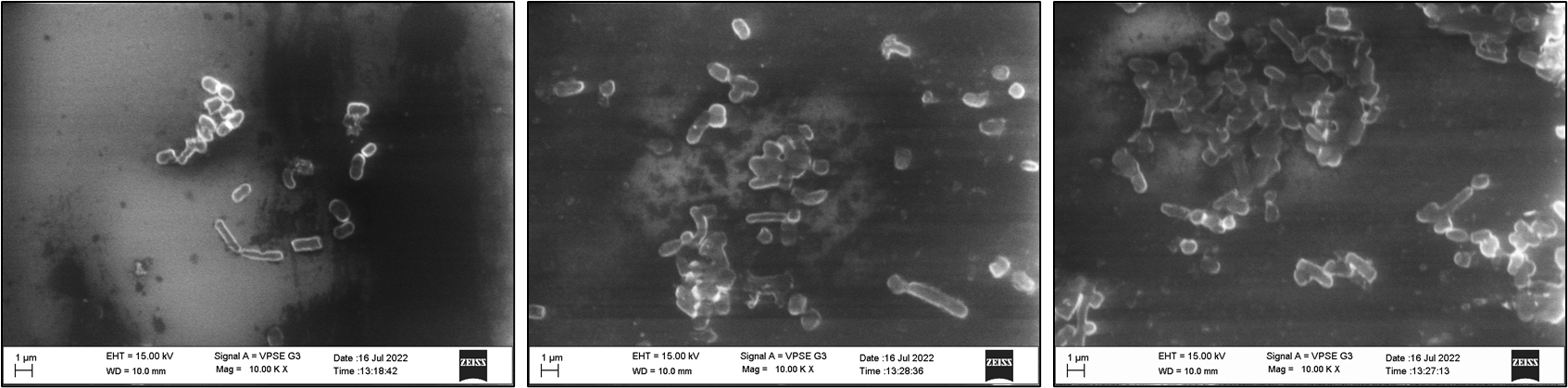
 **Supplementary Figure 1.** Illustrates the SEM images and intricate structural details of *Levilactobacillus brevis* RAMULAB54.


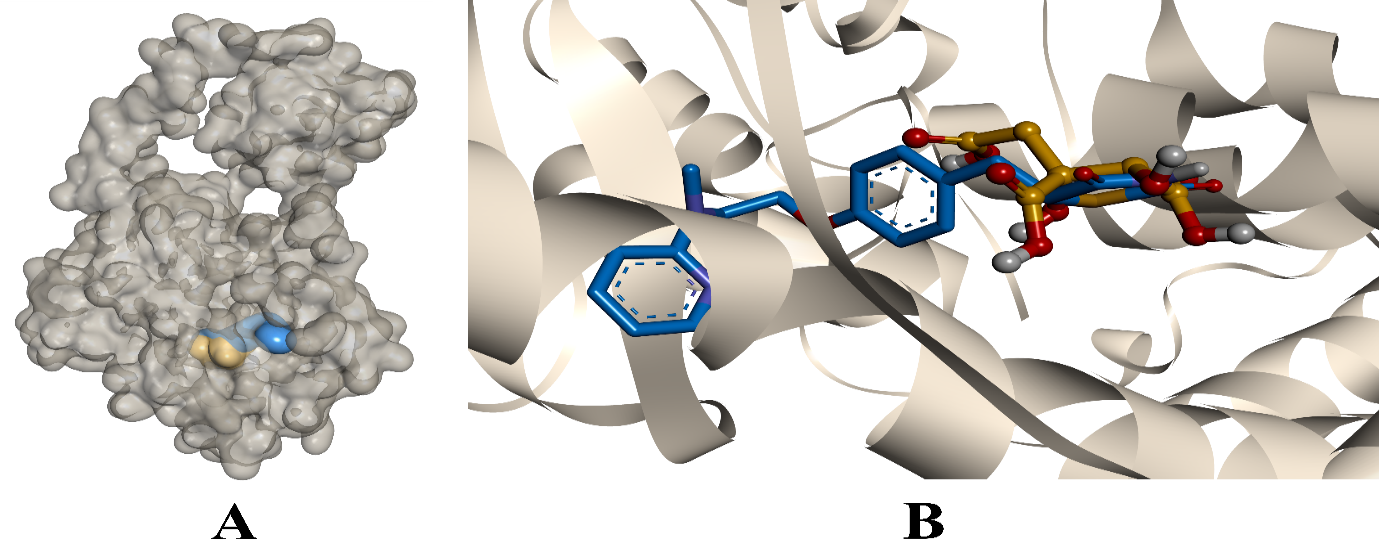
 **Supplementary Figure 2**. Binding of hydroxycitric acid (saffron) and rosiglitazone (blue) inside the PPARγ protein LBD A) Surface representation showing the same binding site of the compounds, B) Ribbon representation showing the alignment and similarity of the binding mode between hydroxycitric acid and rosiglitazone.


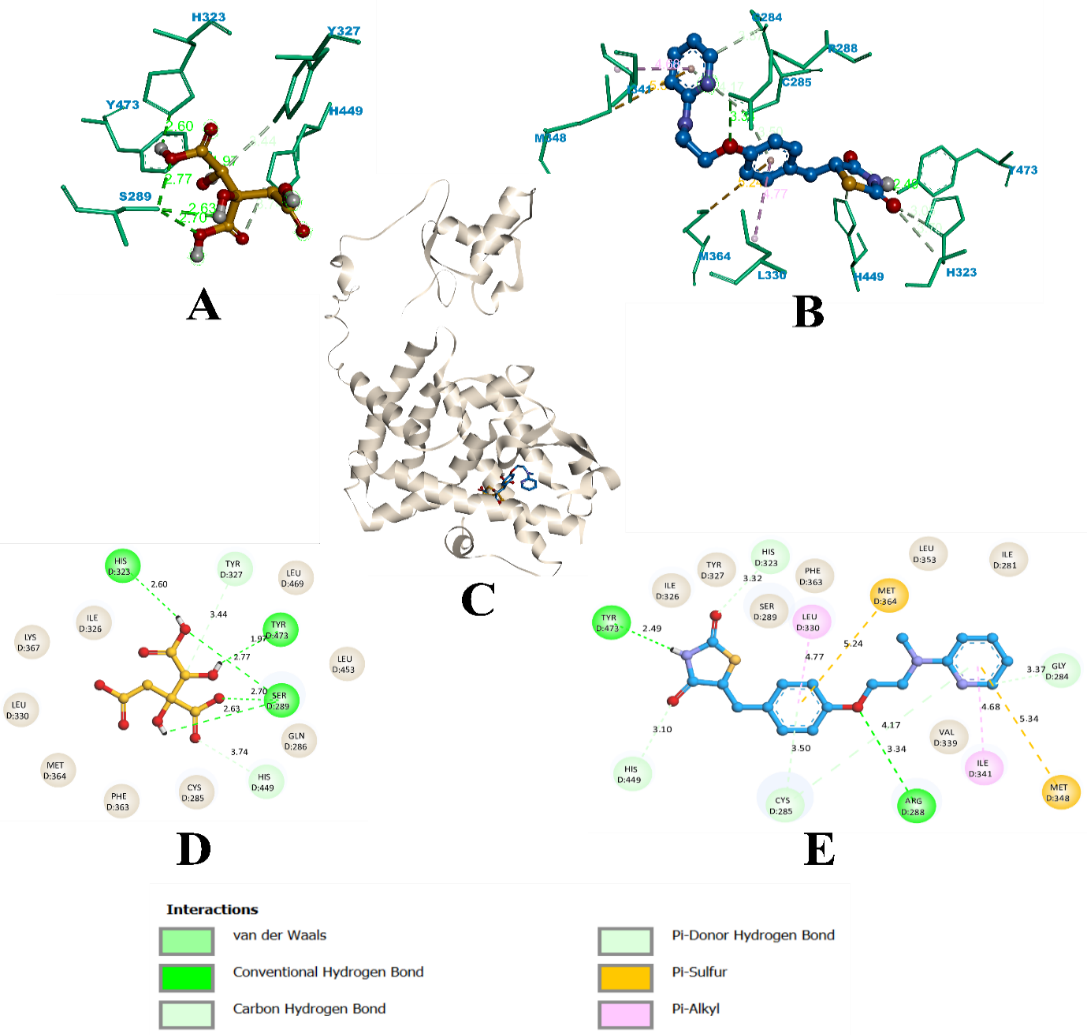


**Supplementary Figure 3.** Binding interactions of hydroxycitric acid and rosiglitazone inside the PPARγ protein LBD A) 3D arrangement of the hydroxycitric acid, B) 2D arrangement of the hydroxycitric acid, C) PPARγ protein LBD showing the binding of both hydroxycitric acid and rosiglitazone, D) 3D arrangement of the rosiglitazone, and E) 2D arrangement of rosiglitazone.


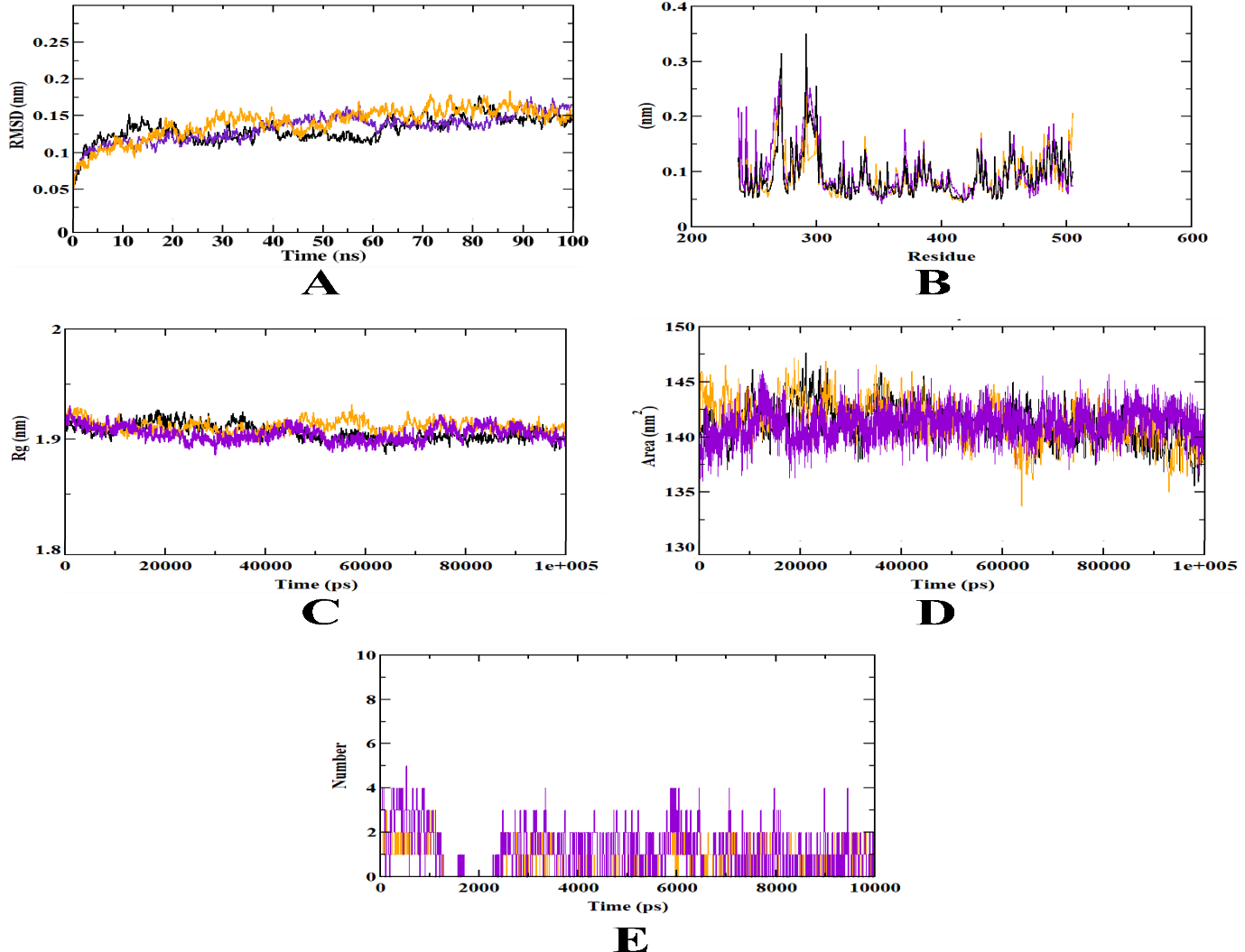


**Supplementary Figure 4**. Molecular dynamics simulation trajectories of hydroxycitric acid and rosiglitazone complexed with PPARγ protein LBD A) RMSD, B) RMSF, C) Rg, D) SASA, and E) Ligand H-Bonds. Saffron: Protein-hydroxycitric acid complex, purple: protein-rosiglitazone complex, black: apo-protein.

 **Supplementary Figure 5:** (A)Effect of LBR54 and Rosiglitazone (RSG) on the cell survival of 3T3-L1 pre-adipocytes after a 24h treatment with increasing doses of each. (B) Effects of LBR54 on lipid accumulation and (C) TG content in 3T3-L1 adipocytes. Results are presented as mean ± SE (n = 3). The significant differences between means at p ≤ 0.05 as assessed by Duncan’s multiple range test.

**Supplementary Figure 6:** Glycaemic response to glucose administration in the Wistar albino rats in an anti-hyperglycaemic study. Each group consisted of n = 15 animals. Data are presented as mean ± SE. Statistical significance was determined using one-way ANOVA followed by DMRT. Comparisons were considered with the superscripts (a–d) significant for p-values ≤ 0.05.

 **Supplementary Figure 7:** Lipid profile of (A) anti-hyperlipidaemic study and (B) antihyperglycemic study treated with ***LB13243***. In the anti-hyperlipidaemic study, each group consisted of **n = 10** animals, while in the anti-hyperglycaemic study, each group consisted of **n = 15** animals. Data are presented as mean ± SE. Statistical significance was determined using one-way ANOVA followed by DMRT. Comparisons were considered with the superscripts (a–d) significant for p-values ≤ 0.05.

**Supplementary Figure 8:** RNA Gene Expression Analysis in Liver and Adipose Tissue of Antihyperlipidemic Groups. qRT-PCR was performed to assess the mRNA expression levels in (A) liver and (B) adipose tissue. Gene expression was normalized to β-actin, and relative expression levels were calculated using the ΔΔCt method. Data are presented as mean ± SE from three independent biological replicates. Statistical significance was determined using one-way ANOVA followed by DMRT, with superscripts (a–d) indicating significant differences (p ≤ 0.05).

**Supplementary Figure 9**: RNA Gene Expression Analysis in Liver, Pancreas, and Adipose Muscle Tissue of Antihyperglycemic Groups. qRT-PCR was performed to assess the mRNA expression levels in (A) liver, (B) pancreas, and (C) adipose muscle tissue. Gene expression was normalized to β-actin, and data are presented as mean ± SE (n = 15). Statistical significance was determined using one-way ANOVA followed by DMRT, with superscripts indicating significant differences (p ≤ 0.05).
